# Human iPSC-derived astrocytes generated from donors with globoid cell leukodystrophy display phenotypes associated with disease

**DOI:** 10.1371/journal.pone.0271360

**Published:** 2022-08-03

**Authors:** Richard Lieberman, Leslie K. Cortes, Grace Gao, Hyejung Park, Bing Wang, Patrick L. Jones, R. Bridge Hunter, John P. Leonard, Robert H. Barker

**Affiliations:** 1 Lysosomal Storage and Metabolic Disease Cluster, Rare and Neurologic Disease Therapeutic Area, Sanofi, Framingham, MA, United States of America; 2 US Early Development, Sanofi, Waltham, MA, United States of America; The University of Texas Rio Grande Valley, UNITED STATES

## Abstract

Globoid cell leukodystrophy (Krabbe disease) is a fatal neurodegenerative, demyelinating disease caused by dysfunctional activity of galactosylceramidase (GALC), leading to the accumulation of glycosphingolipids including psychosine. While oligodendrocytes have been extensively studied due to their high levels of GALC, the contribution of astrocytes to disease pathogenesis remains to be fully elucidated. In the current study, we generated induced pluripotent stem cells (iPSCs) from two donors with infantile onset Krabbe disease and differentiated them into cultures of astrocytes. Krabbe astrocytes recapitulated many key findings observed in humans and rodent models of the disease, including the accumulation of psychosine and elevated expression of the pro-inflammatory cytokine IL-6. Unexpectedly, Krabbe astrocytes had higher levels of glucosylceramide and ceramide, and displayed compensatory changes in genes encoding glycosphingolipid biosynthetic enzymes, suggesting a shunting away from the galactosylceramide and psychosine pathway. In co-culture, Krabbe astrocytes negatively impacted the survival of iPSC-derived human neurons while enhancing survival of iPSC-derived human microglia. Substrate reduction approaches targeting either glucosylceramide synthase or serine palmitoyltransferase to reduce the sphingolipids elevated in Krabbe astrocytes failed to rescue their detrimental impact on neuron survival. Our results suggest that astrocytes may contribute to the progression of Krabbe disease and warrant further exploration into their role as therapeutic targets.

## Introduction

Krabbe disease (globoid cell leukodystrophy) is a rare lysosomal storage disorder characterized by severe demyelination, neurodegeneration, and progressive motor and cognitive deterioration that results from dysfunctional activity of galactosylceramidase (GALC) [1, 2]. Histologically, the brains of Krabbe disease patients are noted for the appearance of multinucleated globoid cells, loss of myelin, and prominent gliosis within the white matter [3]. Deficiency of GALC arises due to mutations in its gene, *GALC* [4–6], leading to impaired degradation of the glycosphingolipid galactosylceramide [7, 8]. Accumulation of galactosylceramide does not directly result in neuropathology, but rather it is its byproduct psychosine (galactosylsphingosine), generated via the lysosomal actions of acid ceramidase, that is thought to be the pathogenic driver of disease [7, 9, 10]. Indeed, higher concentrations of psychosine are observed in the severe early onset form of the disease compared to the less severe late onset forms, and reduced psychosine levels correlate with the therapeutic benefit of allogenic hematopoietic stem cell transplantation [11, 12].

Cytotoxicity of psychosine is mediated through multiple, broad mechanisms. This includes disruption of lysosomal pH [13], perturbation of cellular membrane and lipid raft architecture [14, 15], inhibition of protein kinase C activity [16], generation of toxic lysophosphatidylcholine (LPC) via the actions of phospholipase A2 [17, 18], depolarization of mitochondrial membrane potential [19], and induction of inflammatory cytokines and upregulation of nitric oxide synthase (iNOS) [20, 21]. In the central nervous system, oligodendrocytes are particularly sensitive to impairments of GALC enzyme activity and psychosine exposure, as galactosylceramide is highly expressed in myelinating cells [5, 22]. Since Krabbe disease is hallmarked by robust white matter pathology including demyelination and a near complete loss of oligodendrocytes [23], these cells have been the primary focus of research examining the effects of psychosine on cellular perturbations related to disease progression. However, other neural cell types are sensitive to *GALC* mutations and are negatively affected by elevated psychosine in the disease state, including neurons [24–27], microglia [28–31], and astrocytes [18, 31–34]. Together these results suggest that numerous cell types may be implicated disease pathology, which warrants further investigation into how complex interactions between cells of the brain might be perturbed in Krabbe disease.

In the context of Krabbe disease, GALC deficient murine oligodendrocytes derived from the *twitcher* mouse [35] were able to survive and myelinate axons when implanted into the myelin-deficient *shiverer* mouse [36], suggesting, in part, that non-cell autonomous factors can influence Krabbe oligodendrocyte survival. Indeed, the effects of astrocytes have been implicated in disease pathogenesis through two seminal findings; First, the astrocytic extracellular protease MMP-3 (matrix metalloproteinase-3) is significantly upregulated in the *twitcher* mouse and its expression influences microglia to form the namesake globoid cells observed in Krabbe disease [28]. Second, the enzyme that synthesizes the neuroinflammatory mediator prostaglandin D2 (HPDGS) is increased in *twitcher* microglia, and its receptor, prostaglandin DP1, is upregulated in activated *twitcher* astrocytes. HPDGS inhibition or genetic ablation of the DP1 receptor ameliorated disease phenotypes including astrogliosis, demyelination, and oligodendrocyte apoptosis in the rodent model [37]. Overall, the contribution of astrocytes to the pathology of leukodystrophies is of expanding interest [38], sparked by findings that mutations specifically in astrocyte-expressing glial-fibrillary acidic protein (GFAP) gene cause demyelinating Alexander disease [39], defective support and differentiation of astrocytes results in vanishing white matter disease [40–42], and astrocyte dysfunction precedes demyelination observed in X-linked adrenoleukodystrophy [43], among many others [44, 45]. Therefore, examination of how Krabbe astrocytes impact neurons and microglia warrant further study, particularly in the setting of human Krabbe patient-derived cells.

iPSCs generated from human fibroblasts [46] offer the ability to differentiate patient-derived, disease-relevant cell types. While the murine model of Krabbe disease recapitulates aspects of the human condition [35], and iPSCs have been generated from these mice for differentiation into disease-specific cell types *in vitro* [47], generation of human-derived cell types relevant to Krabbe disease may provide valuable insight into novel mechanisms related to disease progression. For example, a recent study demonstrated GALC-dependent psychosine accumulation and defective differentiation of human iPSCs into a mixed population of neurons, astrocytes, and oligodendrocyte precursors [48]. Additional studies are needed to elucidate cellular mechanisms implicated in neuropathology, including experiments designed to examine effects of GALC mutations of specific cell types, including astrocytes.

To this end, the goal of the current study was to explore Krabbe-associated phenotypes in human iPSC-derived astrocytes. The ability of iPSC-derived astrocytes to recapitulate key findings observed in Krabbe human and mouse studies was validated. Perturbations in lipid levels and lipid biosynthesis genes were observed. Methods to co-culture astrocytes and neurons and astrocytes and microglia of different genetic backgrounds were established and used to identify disease-relevant phenotypes. Finally, this culture system allowed us to explore the utility of substrate reduction approaches that aim to normalize perturbed lipid levels. Overall, the results of our study add to the existing understanding of Krabbe disease and further implicate astrocytes as a contributing cell type to Krabbe disease progression.

## Materials and methods

### Generation of iPSCs

Cells derived from three healthy controls and two donors with infantile onset Krabbe disease were obtained from commercial vendors for reprogramming into iPSCs. Details for the cell lines are as follows: Control 1 (Coriell, GM05659, 1 year old male, Caucasian), Control 2 (Lonza, CC-2511, 31 year female, Caucasian), Control 3 (Icelltis, HUCMF05, male cord blood), Krabbe 1 (Coriell, GM06806, 2 year female, Caucasian), and Krabbe 2 (Coriell, GM01773, 1 year male, Caucasian). Both Krabbe fibroblasts lines were homozygous for a ~30 kb deletion in *GALC* resulting in the loss of exons 11–17, which is the most common mutation associated with Krabbe disease in Caucasians and results in a severe infantile onset form of the disease [49]. Quantitative polymerase chain rection (qPCR) using TaqMan probe and primer sets designed for specific intron/exon boundaries confirmed the genetic characterization of the cell lines. No detectable expression of *GALC* mRNA was observed between exons 13–16 in the Krabbe fibroblasts (S1 Fig).

Reprogramming to pluripotency was performed using the CytoTune 2.0 sendai virus kit (Thermo-Fisher Scientific) to overexpress four transcription factors (OCT4, SOX2, KLF4, and c-Myc) following the manufacturer’s protocol. Individual colonies that formed following viral transduction were manually selected and expanded as clones on Matrigel (Corning) coated plates using mTeSR1 culture medium (StemCell Technologies). One clone was used per donor subject. Expression of pluripotency markers OCT4 and NANOG were validated using immunocytochemistry. G-banded karyotyping was performed by the WiCell Institute (Madison, WI). All iPSC lines used in the current study displayed a normal karyotype.

### Differentiation of neurons and astrocytes

Neural progenitor cells (NPCs) were differentiated from heathy control and Krabbe iPSCs using a dual-SMAD inhibition approach [50, 51]. When iPSCs grown on Matrigel coated plates reached ~20–30% confluence, medium was switched to neural induction medium 1 containing a 50/50 mixture of Advanced DMEM/F12 (Thermo-Fisher Scientific) and Neurobasal (Thermo-Fisher Scientific) supplemented with 1x N2 Supplement (Thermo-Fisher Scientific), 1X B27 Supplement (Thermo-Fisher Scientific), 1X GlutaMAX (Thermo-Fisher Scientific), 10 ng/mL human leukemia inhibitory factor (Millipore), 3 μM CHIR99021 (Stemgent), 2 μM SB432542 (Stemgent), 2 μM Dorsomorphin (StemCell Technologies), and 0.1 μM Compound E (StemCell Technologies). Medium was fully replaced daily for 2 days, following which cells were switched to neural induction medium 2, which was the same as described above without the addition of 2 μM Dorsomorphin, for an additional 5 days, with medium replaced daily. NPCs were dissociated using Accumax (Millipore), counted, and 2.5 million cells were seeded onto Matrigel (Corning) coated 10 cm dishes in neural induction maintenance medium (as describe above without the additions of 0.1 μM Compound E and 2 μM Dorsomorphin). Maintenance medium was replaced daily, and NPCs were passaged onto fresh Matrigel coated dishes using Accumax every 5–7 days when they reached confluency. After 2–3 passages, NPCs were cryopreserved in mFreSR (StemCell Technologies) for subsequent differentiation into neuron and astrocyte cultures. Successful differentiation of NPCs was validated using immunocytochemistry for Nestin and SOX2.

Neuronal differentiation was performed using an established protocol that generates electrophysiologically-active neurons enriched for tyrosine hydroxylase positive dopamine neuron subtypes [51]. NPCs from the healthy controls and Krabbe donors were seeded onto Matrigel coated 10 cm dishes at a density of 800,000 cells per plate in neural induction maintenance medium supplemented with 5 μM Y-27632 (StemCell Technologies). The following day, medium was aspirated and replaced with neural differentiation medium 1 consisting of DMEM/F12 supplemented with 1X N2 Supplement, 1X B27 Supplement, 1X penicillin-streptomycin, 300 ng/mL cAMP (Sigma-Aldrich), 2 mM vitamin C (Sigma-Aldrich), 100 ng/mL sonic hedgehog (SHH, R&D Systems), and 100 ng/mL FGF-8b (Peprotech). Medium was fully replaced every other day for 11 days. On Day 11, medium was aspirated and replaced with neural differentiation medium 2, consisting of DMEM/F12 supplemented with 1X N2 Supplement, 1X B27 Supplement, 1X penicillin-streptomycin, 300 ng/mL cAMP, 2mM vitamin C, 500 μM dibutyryl cAMP (Sigma-Aldrich), 10 ng/mL of each brain-derived neurotrophic factor (BDNF, R&D Systems), glial-derived neurotrophic factor (GDNF, R&D Systems), and insulin-like growth factor-1 (IGF-1, Peprotech), 1 ng/mL TGF-β3 (R&D Systems), and 1 μg/mL laminin (Sigma-Aldrich). Medium was replaced every other day for 5 days. On Day 16 (D16) neuronal cultures were dissociated using Accumax and cryopreserved in complete neural differentiation medium 2 supplemented with 10% DMSO. Characterization of neural cultures by immunocytochemistry on Day 23 revealed expression of pan-neuronal markers HuC/D, Tuj1, and MAP2 (S2A Fig). Quantification revealed ~ 80% HuC/D+ terminally differentiated neurons and ~ 20% SOX2+ NPCs (S2B Fig). No robust difference was observed in the morphology of control and Krabbe TUJ1+ and MAP2+ neurons. Consistent with the published protocol [51], our neuronal cultures contained a subset of tyrosine hydroxylase positive dopamine neurons, highlighted in orange in the middle row of S2A Fig.

Astrocytes were differentiated following previously described methods [52] that utilize the “spontaneous emergence approach” [53], whereby extended culture of mixed neurons and NPCs leads to the emergence of astrocytes that can be expanded. Day 16 cryopreserved neuron/NPCs were thawed onto poly-L-ornithine (10 μg/mL, Sigma-Aldrich) and laminin (10 μg/mL, Sigma-Aldrich) coated 10 cm dishes at a density of ~ 2.5 million cells per plate in NDM-2 medium supplemented with 5 μM Y-27632. The following day medium was replaced with NDM-2 without the addition of Y-27632. 50% medium changes were performed every 3 days for an additional 21 days to allow for the spontaneous generation of astrocytes. On Day 38 of differentiation, cells were dissociated using Accumax and plated 1:2 onto poly-L-ornithine and laminin coated 10 cm dishes in astrocyte differentiation medium consisting of Advanced DMEM/F12 supplemented with 1X B27 Supplement, 1X penicillin-streptomycin, 1X GlutaMAX, 1% FBS (Thermo-Fisher Scientific), and 2 μg/mL Heparin (Sigma-Aldrich). Astrocyte differentiation medium (ADM) was fully replaced three times per week. Astrocytes were expanded in ADM until confluency (~7 days) and passaged 1:3 until Day 63–65 (D63-65), following which astrocyte cultures were dissociated with Accumax and cryopreserved in ADM supplemented with 10% DMSO.

### Differentiation of microglia

Microglia-like cells were differentiated from Control 1 and Control 3 donors as described [54–56]. Embryoid bodies (EBs) were generated from confluent iPSC cultures by dissociating them to single cells and seeding at a density of ~4 x 10^6^ cells per well of Aggrewell 800 6-well plates (StemCell Technologies) in EB medium consisting of complete mTeSR1 supplemented with 10 μM Y27632 (StemCell Technologies), 50 ng/mL BMP-4, 20 ng/mL stem cell factor (SCF), and 50 ng/mL VEGF-121 (all from Peprotech). EB medium was replaced every other day for 7 days. EBs were collected from the Aggrewell plates, passed through a 40 μm filter, centrifuged, and resuspended in hematopoietic cell medium consisting of X-VIVO 15 medium (with gentamicin and phenol red) supplemented with 2 mM Glutamax, 1X penicillin/streptomycin, 55 μM β-mercaptoethanol (Gibco), 100 ng/mL M-CSF (Peprotech), and 25 ng/mL IL-3 (Cell Guidance Systems). EBs were seeded into tissue culture treated flask at a density of 150 EBs per T150 flask. An equal volume of fresh hematopoietic cell medium was added to the cultures on Days 7 and 14 following seeding, with no medium being removed. Thereafter, ~ 75% of the medium was collected and replaced with fresh hematopoietic cell medium every 7 days. Primitive macrophage precursors (PMPs) were harvested during the medium change by collecting supernatant and passing it through a 40 μm filter. Cells were centrifuged and resuspended in microglia medium consisting of Advanced DMEM/F12 supplemented with 2mM Glutamax. 1X penicillin/streptomycin, 1X N2 supplement, 100 ng/mL IL-34, and 10ng/mL GM-CSF (both from Peprotech). PMPs were seeded into tissue culture treated 15 CM plates at 7.5 million cells/plate for terminal differentiation and fresh microglia medium was fully replaced every 2–3 days for 7 days, following which microglia were removed from the plate by incubating with Accumax and a lifting with a cell scraper. Microglia were collected, centrifuged, and frozen in complete microglia medium supplemented with 10% DMSO. Successful differentiation was confirmed using antibodies against IBA1 and CD11b.

### Establishment of co-culture models and compound treatment

Cryopreserved astrocytes were seeded onto poly-L-ornithine (10 μg/mL, Sigma-Aldrich) and laminin (10 μg/mL, Sigma-Aldrich) CellCarrier Ultra high content imaging plates (PerkinElmer) in complete astrocyte differentiation medium at 10,000 cells/well in 96-well format. Cells were cultured to confluency for 3–7 days prior to establishment of co-culture. Astrocyte medium was aspirated and cryopreserved neurons generated from the Control 1 or Control 2 donor were seeded in NDM-2 at a density of 60,000 cells/well. Cryopreserved microglia generated from the Control 1 or Control 3 donor were seeded in complete microglia medium at a seeding density of 60,000 cells/well. Co-cultures were maintained for 96 hours prior to medium aspiration and fixation in 4% paraformaldehyde for immunocytochemistry analysis described below.

To examine the effects of glucosylceramide synthase (GCS) and serine palmitoyltransferase (SPTLC1) inhibition on neuron cell count in the co-culture paradigm, cryopreserved astrocytes were first seeded onto poly-L-ornithine (10 μg/mL, Sigma-Aldrich) and laminin (10 μg/mL, Sigma-Aldrich) 96-well CellCarrier Ultra high content imaging plates (PerkinElmer) in complete astrocyte differentiation medium. The following day, medium was fully replaced with astrocyte differentiation medium containing the indicated concentration of compounds. The GCS inhibitor in the current study was an internal compound and used at its IC99 of 18nM. The SPTCL1 inhibitor Myriocin was purchased from Sigma-Aldrich and used at a range of concentrations (100 nM, 1 μM and 10 μM). Compounds were applied to astrocyte mono-cultures for 3–6 days prior to establishment of neuron co-culture, with 50% of medium replaced after 3 days. For co-cultures, astrocyte medium was aspirated and replaced with complete NDM-2 medium containing 2X each compound. 60,000 control donor-derived neurons were then seeded directly in an equal volume of NDM-2 to dilute compounds to working concentrations. Co-cultures were maintained for 3–4 days prior to assay via immunocytochemistry.

### Astrocyte conditioned media effect on neuron viability

Cryopreserved astrocytes from Control and Krabbe donors were seeded onto poly-L-ornithine (10 μg/mL, Sigma-Aldrich) and laminin (10 μg/mL, Sigma-Aldrich) 96 well CellCarrier Ultra high content imaging plates (PerkinElmer) in complete astrocyte differentiation medium at 10,000 cells/well. Following 3 weeks of culture, with 50% astrocyte medium replaced twice per week, medium was fully replaced with complete NDM-2 and conditioned for 96 hours. At this time, cryopreserved neurons from the Control 2 donor were seeded at 10,000 cells/well onto a poly-L-ornithine (10 μg/mL, Sigma-Aldrich) and laminin (10 μg/mL, Sigma-Aldrich) 384-well CellCarrier Ultra high content imaging plate (PerkinElmer) in NDM-2. Following 96-hours of culture, astrocyte conditioned medium was collected and medium from the neurons was aspirated and replaced with the astrocyte conditioned medium. After 96-hours of exposure to astrocyte conditioned medium, neuron viability was quantified by incubating with Calcein Green AM, NucRed Dead, and Hoechst fluorescent probes in PBS +magnesium +calcium for 10 minutes at 37C and imaging on a PerkinElmer Opera Phenix microscope. Cells were imaged with a 20X objective and viability was calculated as the percentage of Calcein Green AM positive, NucRed Dead negative nuclei over the total nuclei (identified with Hoechst) using associated Perkin Elmer Harmony software.

### Stimulation of astrocytes with TNFα

Astrocytes from two control and two Krabbe donors were thawed and seeded into a poly-L-ornithine (10 μg/mL, Sigma-Aldrich) and laminin (10 μg/mL, Sigma-Aldrich) coated 96 well plate in complete astrocyte medium and cultured for 7 days, with 50% medium changed after 3 days. Astrocyte cultures were treated with 50 ng/mL TNFα (Sigma-Aldrich) diluted in complete astrocyte medium, or mock medium as a control. Cells were incubated with TNFα for 24 hours, following which medium was aspirated and cells were harvested for RNA expression via qPCR using the Fast Cells-to-Ct kit (Thermo-Fisher Scientific) as described below.

### Immunocytochemistry

Mono-cultures and co-cultures of cells from Control and Krabbe donors were fixed in 4% paraformaldehyde for 15 minutes at room temperature, washed twice with PBS, and blocked and permeabilized in 10% normal goat serum (Thermo-Fisher Scientific) (or donkey serum (Sigma-Aldrich) if a goat-derived primary antibody was used) supplemented with 0.3% triton X-100 (Sigma-Aldrich) for 15 minutes at room temperature. Following aspiration of blocking solution, primary antibodies were added in antibody dilution buffer consisting of 2% normal goat serum (or donkey serum (Sigma-Aldrich) if a goat-derived primary antibody was used), 1% bovine serum albumin (Sigma-Aldrich) and 0.1% TWEEN-20 (Sigma-Aldrich) in PBS. The following primary antibodies were used: goat anti-IBA1 (1:500, Abcam Ab5076), mouse anti-HuC/D (1:100, Thermo-Fisher Scientific A21271), rabbit anit-MAP2 (1:500 Ab32454), mouse anti-Tuj1 (1:2000, Millipore MAB1637), rabbit anti-SOX2 (1:500, Millipore, Ab5603), mouse anti-CD44 (1:500, BD Biosciences 550392), rabbit anti-S100β (1:100, Abcam Ab52642), mouse anti-vimentin (1:1000, Sigma-Aldrich V2258), and mouse anti-GLAST (1:100, Abcam Ab416). Primary antibodies were incubated overnight at 4˚C, following which cells were washed four times in PBS and donkey anti-rabbit 488, donkey anti-mouse 568, or donkey anti-goat 568 Alexa Fluor-conjugated secondary antibodies (diluted 1:1,000 in antibody dilution buffer; Thermo-Fisher Scientific) were applied at room temperature for 2 hours. Cells were washed four times with PBS and counterstained with Hoechst 33342 (1:10,000; Thermo-Fisher Scientific) and CellMask Deep Red (1:10,000; Thermo-Fisher Scientific) diluted in PBS for 10 minutes at room temperature, following which cells were washed two times with PBS and imaged on a Perkin Elmer Opera Phenix microscope. Quantification of cells in co-culture was performed on the associated Perkin Elmer Harmony software to examine the total number of Hoechst positive nuclei that co-stained with either HuC/D (for neurons) or IBA1 (for microglia) per field of view. Images were acquired with a 20X objective, and 9 fields of view were imaged in 3–6 replicate wells per condition, per donor subject, for quantification.

### Analysis of sphingolipids

Quantitative analysis of sphingolipids was performed by using liquid chromatography with tandem mass spectrometry analysis (LC-MS/MS). To extract ceramide, glucosylceramide, galactosylceramide, and psychosine, an extraction solution containing 0.2% Formic Acid; 5mM Ammonium formate in (50:50) Methanol:Acetonitrile (v/v) with internal standards was used. D35 labeled C18 galactosylceramide (Matreya), C12 ceramide, and D5 labeled psychosine (Avanti Polar Lipids) were used for internal standards. Ceramide, glucosylceramide, OH-glucosylceramide, galactosylceramide, and OH-galactosylceramide were separated using a Thermo Fisher Scientific LX-2 Multiplex system (ThermoFisher Scientific) and Waters Cortecs HILIC column (2.1 mm x 100 mm, 2.7 μm particles, Waters Corp.) and analyzed by an API 5000 triple quadrupole mass spectrometer in MRM mode (Applied Biosystems). Psychosine were separated using a waters Acquity UPLC system and BEH HILIC column (2.1 mm x 100 mm, 1.7 μm particles, Waters Corp.) and analyzed by an API 6500 triple quadrupole mass spectrometer in MRM mode (Applied Biosystems). Calibration curves were generated with C16 ceramide, C16 glucosylceramide, C16 galactosylceramide, and psychosine standards (Avanti Polar Lipids, Alabaster, AL).

To examine basal levels of lipids, D16 neurons and D63-65 astrocytes from 2 healthy controls and 2 Krabbe donors were seeded from cryopreservation onto poly-L-ornithine (10 μg/mL, Sigma-Aldrich) and laminin (10 μg/mL, Sigma-Aldrich) coated 24 well plates at a density of 400,000 cells per well for neurons and 200,000 cells/well for astrocytes. To allow comparison between cell types and to control for potential medium effects all cells were seeded and cultured in complete NDM-2 described above. Cells were maintained in culture for 7 days, then were lifted from the plate with Accumax (Millipore), counted, and pelleted via centrifugation. Cell pellets were stored at -80˚C until lipid extraction. Extraction was performed proportional to cell number at a ratio of 10 μL extraction solution / 10,000 cells to normalize to cell number. Extraction solution (described above) was added to cell pellets, vortexed for 3 minutes at 1500 RPM, centrifuged for 5 minutes at 1000 RPM, and then transferred to a 384 well plate for analysis.

To examine lipid levels following compound treatments described above, on the day of takedown medium was aspirated from astrocytes cultured in 96 well plates and replaced with PBS containing Hoechst 33342 (1:1000, Thermo-Fisher Scientific) and Calcein Green AM (50 μg vial resuspended in 50 μL DMSO, 1:1000, Thermo-Fisher Scientific) for 10 minutes at 37˚C. Cells were washed 3X with PBS and the entire well imaged using a 5X objective on a Perkin Elmer Opera Phenix high content microscope. The number of Calcein Green AM positive viable nuclei was quantified using Perkin Elmer Harmony software. After imaging, PBS was aspirated and cells were dried in a cell culture hood for ~ 2 hours prior to storage in -80˚C until lipid extraction. Lipids were extracted by adding 150 μL extraction buffer (described above) per well, incubating for 5 minutes at room temperature, spinning down the plate and then transferring the extract to a 384 well plate for analysis.

### Gene expression analysis via qPCR

RNA was harvested from astrocytes cultured in 96 well plates using a Fast Cells-to-Ct kit (Thermo-Fisher Scientific, #4399003), per the manufacturer’s instructions. Briefly, medium was aspirated and cells were lysed with 50 μL lysis buffer supplemented with DNAse for 10 minutes with agitation, followed by incubation with 5 μL stop solution for two minutes, with agitation. Lysate was stored at -80˚ until use. 50 μL of cDNA was synthesized using 10 μL input cell lysate mixed with reverse transcription buffer and enzyme mix, per the kit instructions. Reverse transcription cycles were as follows: 37˚C for 60 minutes followed by 95˚C for 5 minutes. Two μL of cDNA was used as input into a 10 μL real time PCR reaction consisting of 0.5 μL each FAM- and VIC-labeled TaqMan probe and primer sets, 5 μL Fast Advanced real time Master Mix (Thermo-Fisher Scientific), and 2 μL nuclease free water. Real time PCR was performed on an Applied Biosystems QuantStudio 7 instrument with the following cycles: 95˚C for 20 sec, followed by 40 cycles of 95˚C for 3 sec and 60˚C for 30 sec. The following TaqMan probes were used in our study (all from Thermo-Fisher Scientific): FAM-labeled *IL6* (Hs00174131_m1), *IL8* (*CXCL8*, Hs00174103_m1), *IL1B* (Hs01555410_m1), *CCL2* (MCP-1, Hs00234140_m1), *ASAH1* (Hs00602774_m1), *UGT8* (Hs00409958_m1), *UGCG* (Hs00916612_m1), *GBA* (GBA1, Hs00986836_g1), *RIPK3* (Hs00179132_m1), and *MT1E* (Hs01652848_g1). Gene expression for targets of interest were normalized to VIC-labeled 18s rRNA (Thermo-Fisher Scientific, 4319413E). Relative expression was calculated on the QuantStudio software using the ddCt method with the value 1 being set to the Control 1 donor.

### IL-6 protein analysis via AlphaLISA

IL-6 protein was measured in the supernatant of astrocyte-conditioned medium. Undiluted supernatant was collected following the indicated culture times and analyzed on a PHERAstar plate reader (BMG Labtech) using an AlphaLISA IL-6 kit (Perkin Elmer), per the manufacturer’s instructions. Concentration of IL-6 was calculated based on the standard curve provided in the AlphaLISA kit. Following collection of the supernatant, cells were live imaged with Calcein Green AM and Hoechst to quantify cell number. PBS containing calcium and magnesium supplemented with Hoechst 33342 (1:1000, Thermo-Fisher Scientific) and Calcein Green AM (50 μg vial resuspended in 50 μL DMSO, 1:1000, Thermo-Fisher Scientific) was applied to the cultures for 10 minutes at 37C. Cells were washed 3X with PBS and the entire well was imaged using a 5X objective on a Perkin Elmer Opera Phenix high content microscope. The number of Calcein Green AM positive nuclei were quantified using Harmony software and used to normalize protein expression values to determine IL-6 concentration on a per-viable cell basis.

### Statistical analysis

Statistical analysis was performed using GraphPad Prism (GraphPad Prism version 8.3.1 for Windows, GraphPad Software, San Diego, California USA, www.graphpad.com). Unpaired Student’s t-tests were used to compare basal glycosphingolipids, gene expression, and number of cells in co-culture between Control and Krabbe cells. Statistics were performed on grouped data (i.e. Control vs. Krabbe), but the data is visualized per subject for the reader to observe between-line differences. Gene expression following TNFα stimulation was assessed using two-way ANOVA on pooled values comparing Control and Krabbe donors. Treatment (TNFα) and Genotype (Control vs. Krabbe) were set as the factors for the two-way ANOVA. A Bonferroni’s multiple comparison post-hoc test was performed specifically comparing Control and Krabbe gene expression values following TNFα stimulation. The effects of compound treatment on lipid levels, IL-6 protein expression, and the number of HuC/D+ nuclei was analyzed by two-way ANOVA with a Bonferroni multiple comparison post-hoc test comparing DMSO and treatment conditions within a given cell line. For all tests, statistical significance was determined as p < 0.05, or adjusted p < 0.05 for the Bonferroni multiple comparison’s tests.

## Results

### Krabbe iPSC-derived astrocytes recapitulate findings from rodent and human models of disease

**Fig 1:**
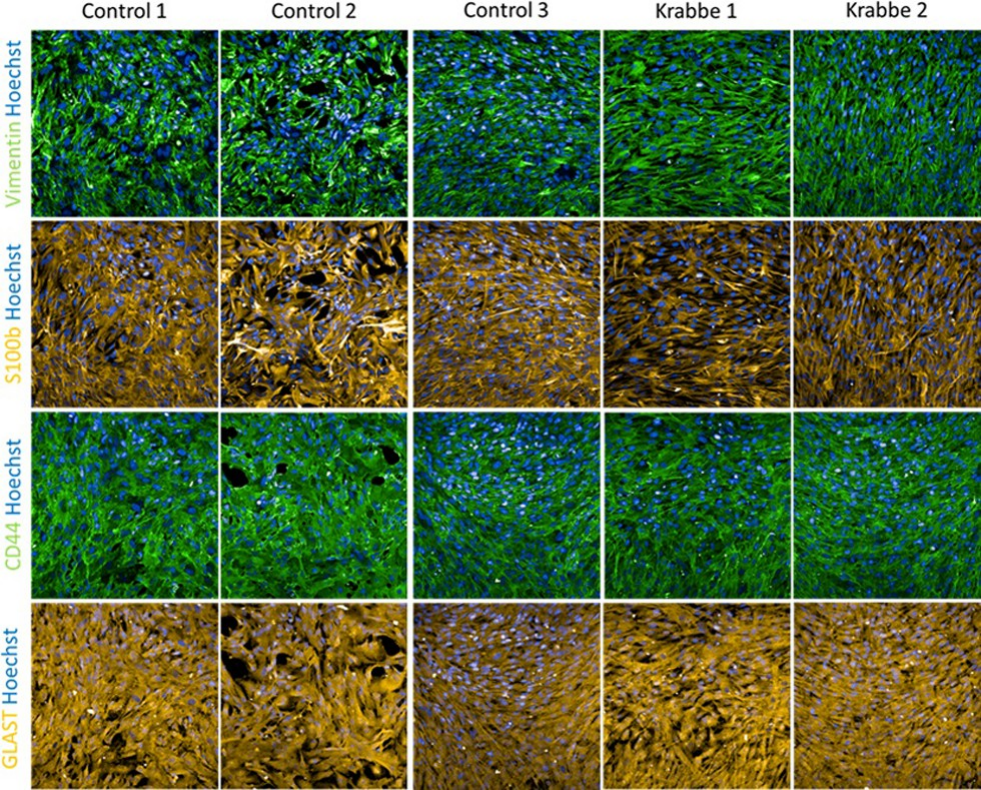
Assessment of differentiation into astrocyte lineage. Representative images of astrocytes differentiated from 3 healthy control donors and 2 donors with infantile onset Krabbe disease caused by a large deletion in the *GALC* gene. Cryopreserved astrocytes were thawed and cultured for 7 days prior to fixation and imaging. Immunocytochemistry revealed positive staining for astrocyte markers vimentin, s100β, CD44, and GLAST (EAAT1) in all cell lines used. Images were taken at 20x magnification.

**Fig 2:**
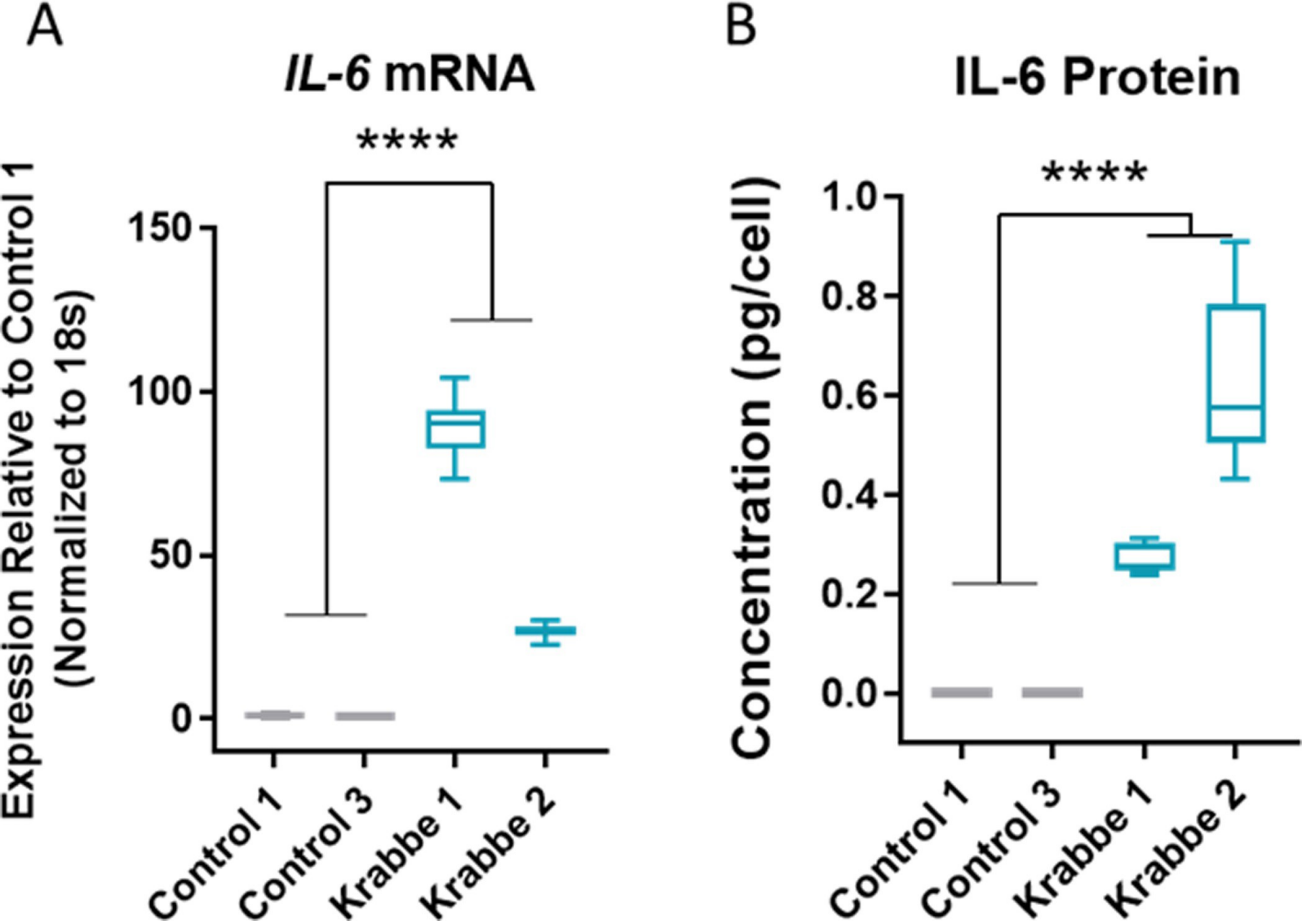
Upregulation of IL-6 in Krabbe astrocytes. Significantly greater IL-6 (A) mRNA and (B) protein expression was observed in astrocytes derived from the donors with Krabbe disease. **** p < 0.0001. Data for individual donor subjects is shown as a box and whisker plot with median represented and errors bars indicating min/max values. Statistical analysis was conducted using t-tests on pooled data comparing control vs. Krabbe cells.

iPSCs from three healthy controls and two donors with infantile onset Krabbe disease successfully differentiated into astrocytes that expressed appropriate lineage markers vimentin, S100β, CD44, and GLAST (Fig 1). No apparent difference in expression patterns were noted between cell lines, and following seeding onto laminin coated plates astrocytes from all donors proliferated and formed confluent monolayers. We validated the utility of our human iPSC model by asking if they recapitulated some established findings in the field. Compared to controls, Krabbe astrocytes had higher expression of *RIPK3* and *MT1E* transcripts (t = 5.8, p < 0.0001, and t = 7.9, p < 0.0001, respectively) (S3 Fig), as previously reported in rodent and human models [57, 58]. While undetectable in the control astrocytes, mRNA and protein expression of IL-6 was detected in unstimulated Krabbe astrocytes (mRNA: t = 7.0, p < 0.0001; Protein: t = 7.3, p < 0.0001) (Fig 2). mRNA expression of *IL-6*, *IL-8*, *IL-1β*, and *MCP-1* (*CCL2*) was profiled following 24-hour TNFα stimulation in control and Krabbe astrocyte cultures. Two-way ANOVA revealed an overall significant effect of TNFα treatment (all p < 0.0001), an overall significant effect of donor genotype (all p < 0.0001, except for *IL-1β*, p < 0.001), and a significant interaction between treatment and genotype (all p < 0.0001, except for *IL-1β*, p < 0.001) for all four gene examined (S4A–S4D Fig), suggesting that Krabbe astrocytes responded differently to TNFα than control astrocytes. Post-hoc analysis identified that Krabbe astrocytes had higher total expression of *IL-6*, *IL-8*, *IL-1β*, and lower expression of *MCP-1 (CCL2)* than controls following TNFα stimulation (all adjusted p < 0.0001) (S4A–S4D Fig). These findings are consistent with those reported in studies examining mechanisms of disease in human and murine models [20, 21, 32, 59]. In sum, these results validated that Krabbe patient-derived iPSCs were able to be differentiated into TNFα-responsive astrocytes that recapitulated some key findings of established models of Krabbe disease.

### Lipid and gene expression perturbations in Krabbe astrocytes

**Fig 3:**
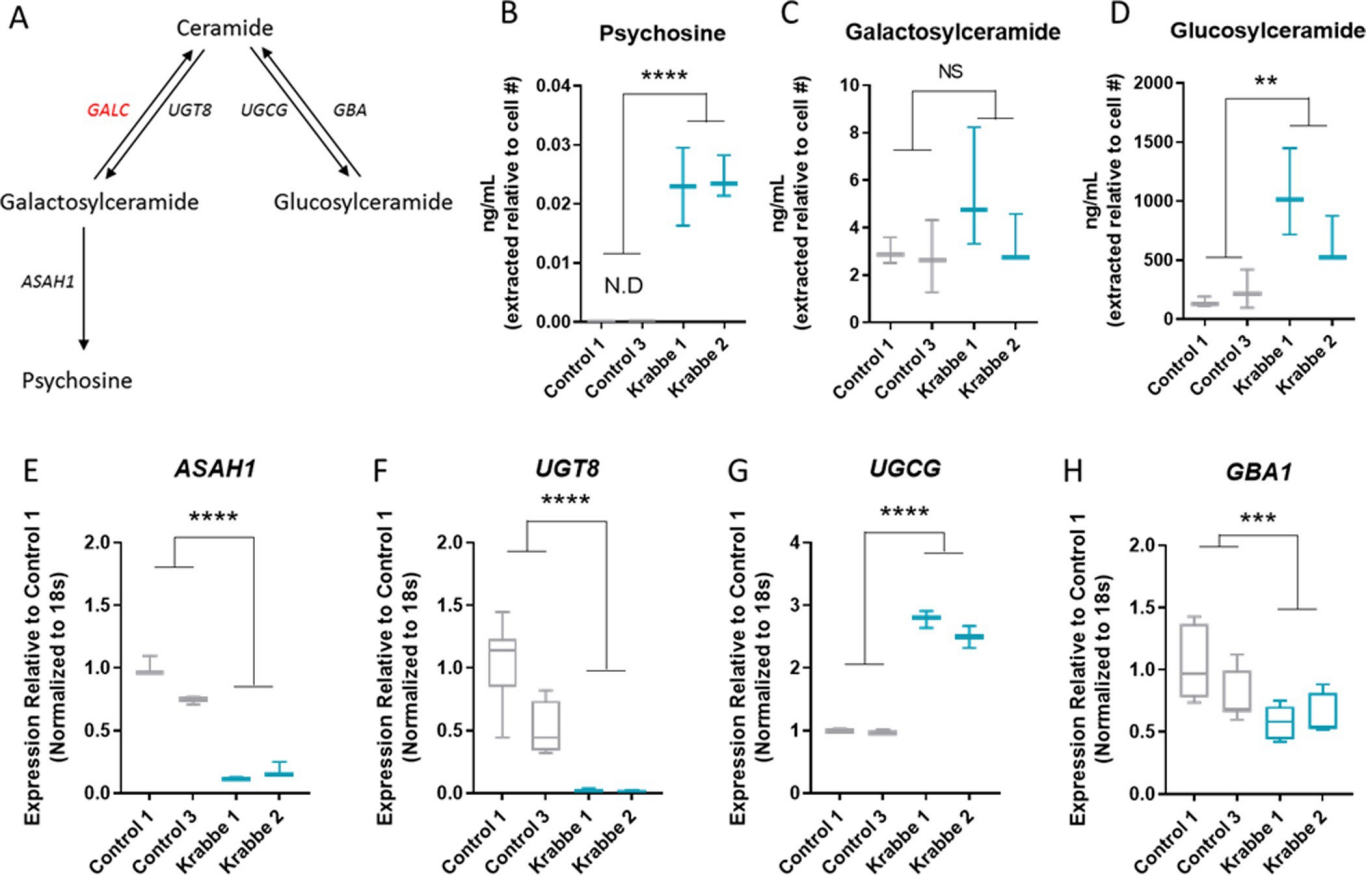
Lipid accumulation and compensatory gene expression changes in Krabbe astrocytes. (A) A simplified schematic of the glycosphingolipid pathway examined in the current study and the genes encoding the enzymes responsible for their biosynthesis. The gene encoding galactosylceramidase (*GALC*), which is defective in Krabbe disease, is indicated in red. (B) Astrocytes generated from the Krabbe donors had significantly elevated levels of psychosine. (C) No significant difference in total non-hydroxylated galactosylceramide was observed between control and Krabbe astrocytes. (D) Increased total non-hydroxylated glucosylceramide was observed in Krabbe astrocytes. (E-H) Compensatory changes in the expression of genes involved in the local glycosphingolipid biosynthesis pathway. For the galactosylceramide arm, a significant downregulation of genes encoding ceramide galactosyltransferase (*UGT8*) and acid ceramidase (*ASAH1*), which are responsible for the biosynthesis of galactosylceramide and psychosine, respectively, was observed in Krabbe astrocytes compared to controls. For the glucosylceramide arm, an upregulation of ceramide glucosyltransferase (*UGCG*) and downregulation of glucocerebrosidase 1 (*GBA1*) was observed in Krabbe astrocytes compared to controls. ** p < 0.01, *** p < 0.001, **** p < 0.0001. Data for individual donor subjects is shown as a box and whisker plot with median represented and errors bars indicating min/max values. Statistical analysis was conducted using t-tests on pooled data comparing control vs. Krabbe cells. N.D. = not detected.

Lipids in the ceramide metabolic pathway relevant to Krabbe disease were profiled by mass spectroscopy (Fig 3A). Psychosine was detectable and elevated in Krabbe astrocytes compared to undetectable levels in control cells (t = 11.0, p < 0.0001) (Fig 3B). No significant difference in total galactosylceramide was observed between control and Krabbe astrocytes (t = 1.6, p = 0.14) (Fig 3C). Examination of specific galactosylceramide isoforms revealed predominant expression of the C16 and C18 chains in our cultures. Krabbe astrocytes had ~2-fold more C16 than controls, while C18 was substantially lower. No other isoforms were robustly detectable (S5 Fig). Total glucosylceramide was significantly elevated in Krabbe astrocytes compared to those derived from healthy controls (t = 4.3, p = 0.002) (Fig 3D). Examination of the specific glucosylceramide isoforms showed this increase was largely driven by longer chain lengths C24:1 and C24 which were ~3-fold higher in Krabbe cells compared to controls. The C16 isoform was also elevated in Krabbe astrocytes, being ~2-fold higher compared to controls (S5 Fig). Hydroxylated galactosylceramide and glucosylceramide were also profiled and were significantly higher in Krabbe astrocytes compared to controls (t = 3.7, p = 0.004, and t = 4.3, p = 0.002, respectively). This increase was driven by the C16 isoforms for both hydroxylated lipids, which was the chain length predominantly expressed in our cells (S6A and S6B Fig).

To complement the mass spectroscopy data, we assessed mRNA expression levels of genes encoding lipid biosynthesis enzymes within the galactosylceramide and glucosylceramide metabolic pathways in control and Krabbe astrocytes (Fig 3A). In the galactosylceramide arm of the pathway, Krabbe astrocytes had significantly lower expression of genes encoding acid ceramidase (*ASAH1*, t = 11.0, p < 0.0001) and ceramide UDP-galactosyltransferase (*UGT8*, t = 7.0, p < 0.0001) (Fig 3E and 3F). In the glucosylceramide arm of the pathway, Krabbe astrocytes had significantly higher expression of glucosylceramide synthase (*UGCG*, t = 18.9, p < 0.0001) and lower expression of glucosylceramidase (*GBA1*, t = 3.7, p = 0.0008) (Fig 3G and 3H). These results are consistent with the mass spectrometry data described above.

We also measured the concentrations of these lipids in iPSC-derived neurons from the same donor subjects, which were generated as part of the astrocyte differentiation protocol (See Materials and Methods). We observed significantly elevated psychosine in Krabbe neurons compared to control neurons (t = 3.5, p < 0.01). There was a slight but significant increase in total non-hydroxylated galactosylceramide in Krabbe neurons (t = 2.4, p = 0.04), but no significant differences in total hydroxylated galactosylceramide (t = 1.8, p = 0.11) or non- hydroxylated and hydroxylated glucosylceramide (t = 1.1, p = 0.29 and t = 1.0, p = 0.34, respectively) (S7A–S7E Fig). While psychosine levels were relatively similar between Krabbe neurons and astrocytes (S7A Fig), we noted that glucosylceramide (both non-hydroxylated and hydroxylated) levels were substantially higher in Krabbe astrocytes compared to donor-matched neurons (S7D and S7E Fig).

### Krabbe astrocytes impact neuron and microglia survival in co-culture

**Fig 4:**
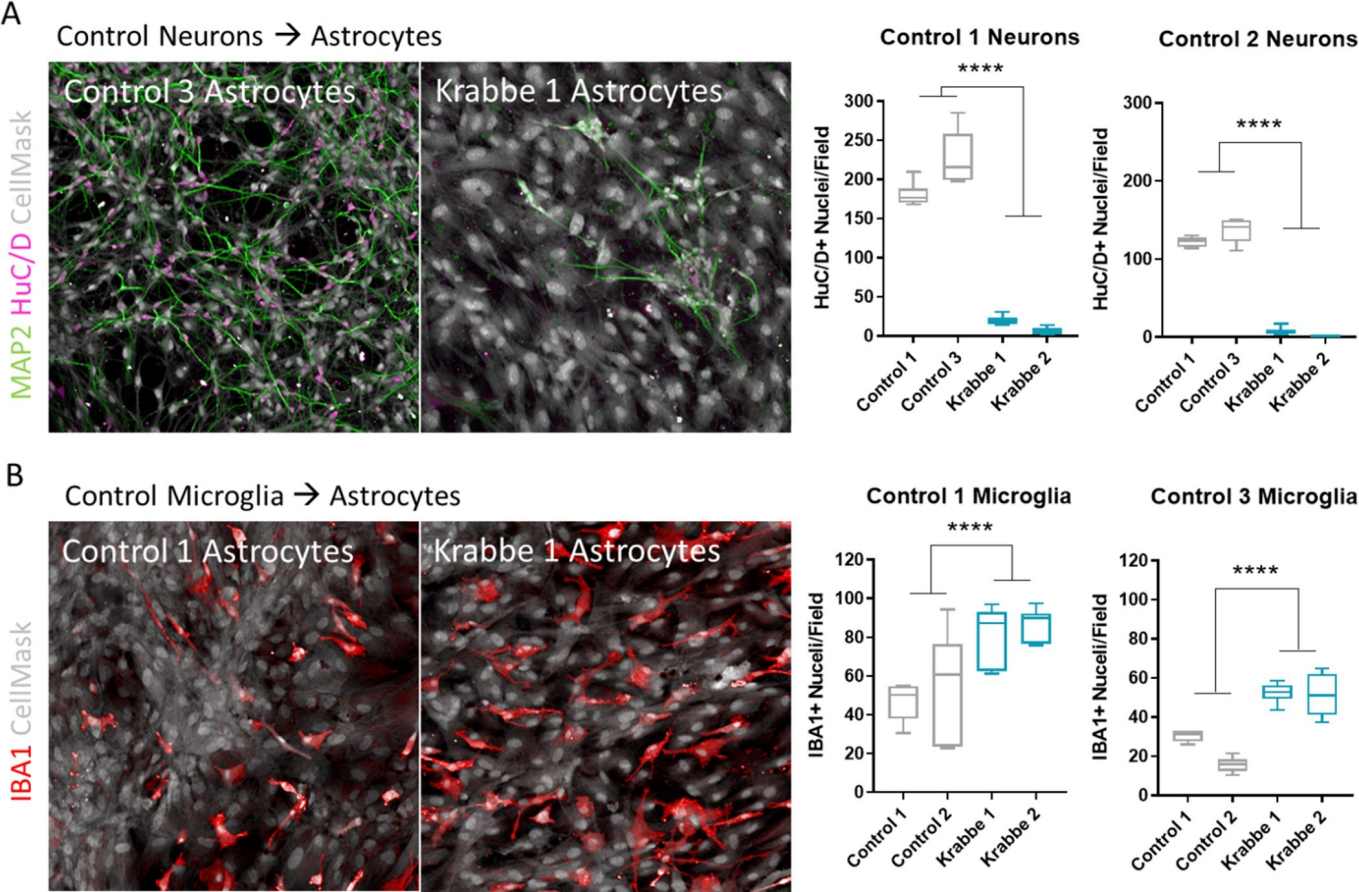
Krabbe astrocytes impact survival of iPSC-derived neurons and microglia in co-culture. Cryopreserved astrocytes from healthy control or Krabbe donors were thawed and cultured for 7 days. Neurons or microglia-like cells generated from two control donors were then seeded onto the established control or Krabbe astrocytes. (A) Representative images of HuC/D positive (purple) and MAP2 positive (green) neurons following 96-hour co-culture with control or Krabbe astrocytes. Significantly fewer HuC/D+ neurons were identified when co-cultured with Krabbe astrocytes. (B) Representative images of IBA1+ (red) microglia following 96-hour co-culture with control or Krabbe astrocytes. A significantly greater number of IBA1+ microglia were identified when co-cultured with Krabbe astrocytes. Representative images in panels A and B were acquired at 20x magnification. **** p < 0.0001. Data for individual donor subjects is shown as a box and whisker plot with median represented and errors bars indicating min/max values. Statistical analysis was conducted using t-tests on pooled data comparing control vs. Krabbe cells.

We established co-cultures of astrocytes-neurons and astrocytes-microglia to explore the impact of Krabbe astrocytes on survival of these cell types. Cryopreserved astrocytes were thawed and cultured for 7 days to form a monolayer prior to the seeding of cryopreserved neurons or microglia derived from control donors. The number of HuC/D+ neurons was significantly lower when co-cultured for 96 hours on Krabbe astrocytes compared to control astrocytes (Control 1 neurons: t = 18.2, p < 0.0001; Control 2 neurons: t = 30.0, p < 0.0001) (Fig 4A). We observed that dense and complex MAP2+ neuronal projections were able to form over this brief time when neurons were co-cultured with astrocytes derived from control donors. Neurons co-cultured on Krabbe astrocytes appeared clustered and MAP2+ neurites were limited. In contrast, the number of IBA1+ microglia were significantly greater when co-cultured for 96 hours on Krabbe astrocytes compared to control astrocytes (Control 1 microglia: t = 4.8, p < 0.0001; Control 3 microglia: t = 8.3, p < 0.0001) (Fig 4B).

As a pilot experiment, we assessed whether direct cell-to-cell contact was needed for the detrimental effect of Krabbe astrocytes on neuronal cell count or if exposure to conditioned media also displayed neurotoxicity. Neuronal medium was conditioned for 96 hours on 3-week-old control and Krabbe astrocyte cultures and then applied to an established mono-culture of Control 2 neurons. Following 96 hours of incubation, neuronal viability was significantly lower in cultures containing conditioned media from Krabbe astrocytes compared to neuronal cultures incubated with conditioned medium from control astrocytes (t = 25.9, p < 0.0001) (S8 Fig).

### Substrate reduction approaches fail to rescue neuronal survival

Given the elevated glucosylceramide we observed in the Krabbe astrocytes, we hypothesized that this elevation could be playing a role in their inability to facilitate neuronal survival. Therefore, we explored whether reducing the elevated glucosylceramide in Krabbe astrocytes would rescue the ability of neurons to survive in a co-culture setting. We reduced glucosylceramide by targeting its biosynthetic enzyme, glucosylceramide synthase (GCS), with a proprietary internal GCS inhibitor (S9A Fig). Treatment with 18 nM (corresponding to the IC 99) of the GCS inhibitor significantly reduced total non-hydroxylated and hydroxylated glucosylceramide in control and Krabbe astrocytes (adjusted p < 0.05 for all lines) (S9B Fig). However, despite a reduction in lipid to non-treated control levels, there was no reduction in basal IL-6 protein expression in supernatant collected from non- TNFα stimulated Krabbe astrocytes (all adjusted p > 0.05) (S9C Fig). To test the effects of astrocytic substrate reduction therapy on neuronal survival, control neurons derived from the Control 2 donor were seeded onto DMSO or GCS inhibitor-treated astrocytes and cultured for 96 hours (S9D Fig). Despite reduced glucosylceramide levels, Krabbe astrocytes negatively impacted neuronal survival, and there was no benefit of glucosylceramide reduction on the number of HuC/D+ nuclei (all p > 0.05) (S9E Fig).

**Fig 5:**
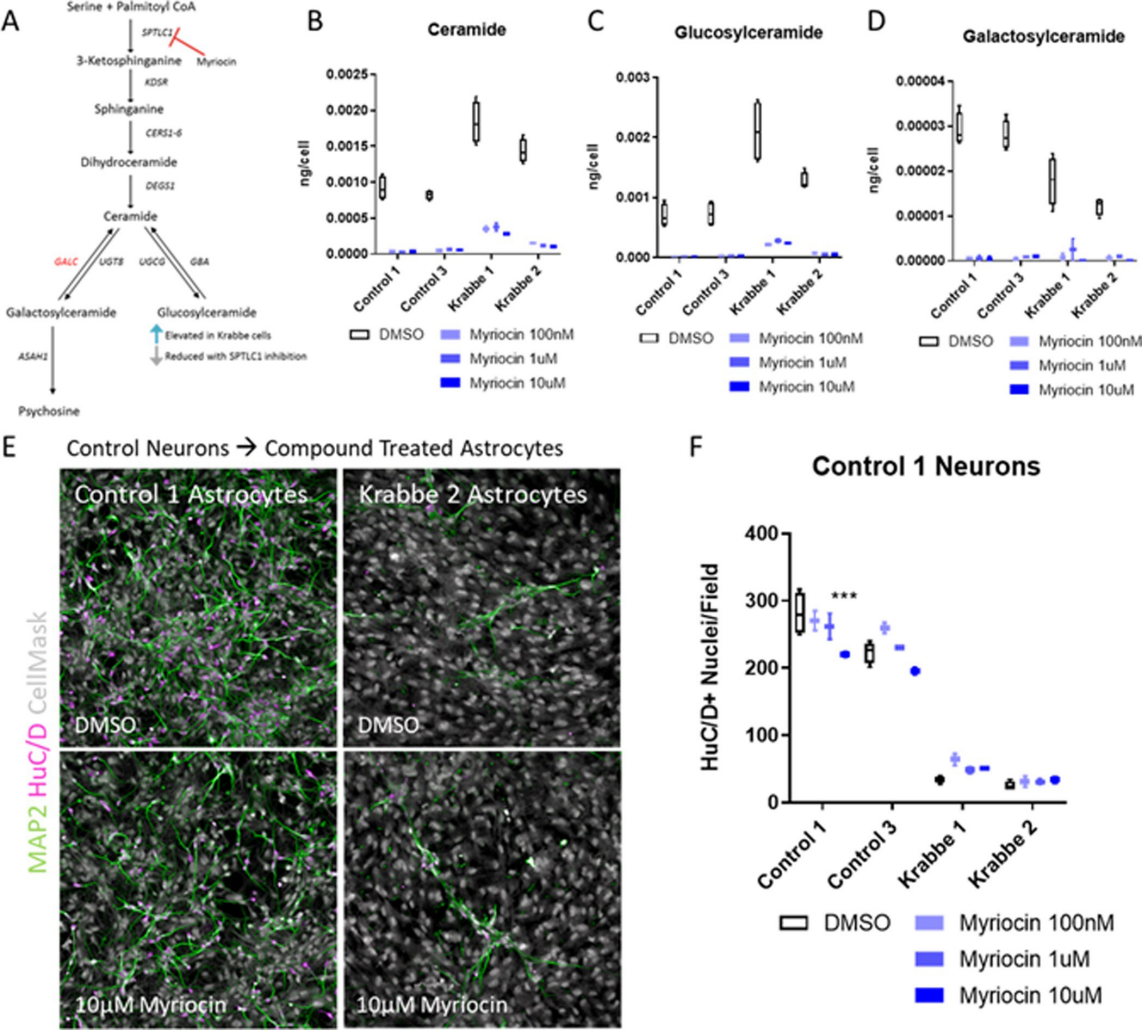
Inhibiting synthesis of ceramide in Krabbe astrocytes fails to facilitate neuronal survival. Cryopreserved astrocytes from healthy control and Krabbe donors were seeded into microwell plates, cultured for 24 hours, and then treated with compound for 6 days prior to analysis. (A) A schematic depicting the biosynthesis of ceramide-derived glycosphingolipids, and the rationale for reducing glucosylceramide via inhibition of SPTLC1 with myriocin. (B-D) Target engagement of myriocin was verified by GC-MS-MS. Myriocin reduced ceramide and its derivatives glucosylceramide and galactosylceramide in control and Krabbe astrocytes. (E, F) Representative images of neuron-astrocyte co-cultures and quantification of the number of HuC/D+ nuclei. No beneficial effect of myriocin was observed when control neurons were co-cultured with Krabbe astrocytes for 72 hours. Data is shown as a box and whisker plot with median represented and errors bars indicating min/max values. *** p < 0.001.

Next, we examined whether reducing total sphingolipids in astrocytes would have a beneficial effect on neuronal survival by inhibiting serine palmitoyltransferase (SPTLC1) with Myriocin, which catalyzes the first step in sphingolipid biosynthesis [60] (Fig 5A). 6-day treatment with Myriocin robustly reduced total levels of ceramide, glucosylceramide (both non-hydroxylated and hydroxylated), and galactosylceramide (both non-hydroxylated and hydroxylated) at all concentrations tested (Figs 5B–5D and S10A and S10B). Interestingly, we noted a significant increase in the basal level of ceramide in Krabbe astrocytes compared to control astrocytes (see Fig 5B, average Control vs. Krabbe t-test: t = 6.6, p < 0.001). Myriocin treatment did not reduce psychosine levels in Krabbe astrocytes and in one line was increased following treatment (adjusted p < 0.05). This could reflect the inability of Krabbe astrocytes to breakdown psychosine existing prior to treatment. Expression of IL-6 protein in the supernatant of Krabbe astrocytes was unchanged in the Krabbe 1 line at all concentrations and in the Krabbe 2 line at 100 nM (adjusted p > 0.05), while slight, but significant, reductions were observed at 1 μM (adjusted p = 0.03) and 10 μM (adjusted p = 0.02) (S11A and S11B Fig). To test the ability of the myriocin treated Krabbe astrocytes to facilitate neuronal survival in co-culture, neurons derived from the Control 1 donor were co-cultured on compound-treated astrocytes for 72 hours (Fig 5E). Two-way ANOVA revealed no significant effect of Myriocin treatment on the number of HuC/D+ nuclei when co-cultured with Krabbe astrocytes (Fig 5F) (all adjusted p > 0.05). We did observe a reduction in HuC/D+ nuclei in the control astrocytes at the highest myriocin concentration tested, which could reflect toxicity of the compound (adjusted p < 0.001). In sum, there was a clear and robust effect of Krabbe astrocytes on neuron survival in co-culture, and this was not changed with either GCS or SPTLC1 inhibition despite validation of the expected reduction in downstream sphingolipids.

## Discussion

In the current study, we generated iPSC-derived astrocytes from a total of three healthy controls and two donors with infantile onset Krabbe disease and used them to explore phenotypes in disease-relevant cell types. Overall, our findings support and complement a previous report examining iPSCs from Krabbe donors by reproducing observed disease-relevant phenotypes, including psychosine accumulation, impairment of neuronal survival, and perturbation of lipid profiles such as the observation of elevated ceramide [48], as discussed in more detail below. Similar to other reports describing astrocyte differentiation, our iPSC-derived astrocytes from control and Krabbe donors expressed the expected markers vimentin, S100β, and CD44, and upregulated cytokine gene expression in response to stimulation with TNFα [52, 61]. Importantly, iPSC-derived astrocytes recapitulated many findings observed in humans and in animal models of the disease, including having higher expression of basal IL-6 transcript and protein [31, 59], elevated mRNA expression of *RIPK3* and *MT1E* [57, 58], as well as higher expression of *IL-6*, *IL-1β*, and *IL-8* and lower expression of *MCP-1* (*CCL2*) following stimulation [20, 21, 32, 59]. Perhaps most importantly with respect to the disease state, astrocytes from the Krabbe donors accumulated psychosine, which was undectable in the contol cells [7]. Of note, it has been reported that exogenous application of psychosine in the micromolar range, a leve that can be observed in patient samples [7], is toxic to astrocytes *in vitro* [32]. In contrast, our Krabbe iPSC-derived astrocytes appeared morphologically similar and grew as expected compared to healthy control astrocytes. We can only speculate that the endogenous accumulation of psychosine observed in our experiments did not reach the toxic levels required for astrocyte toxicicity, which is in concordance with the findings from O’Sullivan and Dev (2015) suggesting that, at optimal density and time of treatment, 10 μM psychosine treatment is toxic, while 5 μM does not impact viability [32]. Future experiments might address if longer time in culture would result in astrocyte growth abnormalities or toxicity, or if the endogenous psychosine might “prime” the astrocytes to be more susceptible to an exogenous challenege. In sum, these results imply the validity of using a human iPSC-derived astrocyte model to explore Krabbe disease biology *in vitro*.

A novel finding of our current study was elevated glucosylceramide in the human Krabbe astrocytes. This was accompanied by compensatory changes in gene expression of the enzymes within the local lipid biosynthesis pathway, with lower *UGT8* and *ASAH1* expression, but higher *UGCG* expression, observed in Krabbe astrocytes compared to controls. These findings indicate a shunting away from galactosylceramide and psychosine and towards glucosylceramide (see schematic in Fig 3A). A recent report by Corado and colleagues [62] was the first to identify elevated glucosylceramide in CSF of a canine model of Krabbe disease, which correlated with psychosine and disease progression. They did not observe significant elevation of glucosylceramide in biopsies of the oligodendrocyte-enriched internal capsule or cerebellar white matter, which might suggest that specific cell types are driving the increase observed in CSF. Although the authors do not examine expression of lipid biosynthesis genes, they speculate in the discussion that broader dysregulation of lysosomal metabolism could lead to a secondary increase in glucosylceramide. The lipid and gene expression results of our study complement their findings and discussion by recapitulating their observed phenotype of elevated glucosylceramide and providing evidence of perturbed dysregulation of the disease-relevant lipid biosynthesis pathway in human cells *in vitro*.

Consistent with the notion of shunting away from the galactosylceramide arm of the pathway, we also observed an increase in total ceramide in Krabbe astrocytes compared to controls. Ceramide has been shown to be elevated in the *twitcher* mouse [63] and in mixed iPSC-derived neural cultures from Krabbe donors [48], providing additional validation of our stem cell model. Furthermore, accumulation of ceramide has been observed in a multitude of degenerative disorders including Alzheimer’s [64, 65], Parkinson’s [66, 67], and amyotrophic lateral sclerosis [68, 69], among others [70], suggesting that accumulation of ceramide may be a commonality among many neurodegenerative diseases including Krabbe disease. Biologically, ceramide contributes to the regulation of apoptosis [71] and inflammation [72], and in the context of disease, accumulation of ceramide may result in increased susceptibility to apoptosis [73] and enhanced neuroinflammation [74, 75]. To our knowledge, no studies have specifically examined the role of astrocytic ceramide in the pathogenesis of Krabbe disease, but in studies of Alzheimer’s disease, aberrant ceramide expression is observed in astrocytes [76], correlates with neuroinflammation [77], and can be secreted in exosomes to influence apoptosis in surrounding cells [78]. With respect to our current study, these results suggest that future research should explore astrocyte-specific sphingolipid metabolism in Krabbe disease. A limitation of the current study is that we did not measure ceramide in the earlier Day 16 neuron cultures, and therefore do not know whether accumulation of ceramide precedes the accumulation of glucosylceramide in Krabbe astrocytes. Taken all together with results of prior studies, our lipid analysis results suggest that the contributions of perturbated sphingolipid biosynthesis to Krabbe disease pathogenesis should continue to be explored.

An exploratory aspect of our study was the in-depth profiling of isoforms of non-hydroxylated and hydroxylated galactosylceramide and glucosylceramide (S5 and S6 Figs) that arise due to various fatty acid chain lengths and hydroxylation state [79]. We observed that shorter chain length (C:16 and C:18) non-hydroxylated galactosylceramides were prominent in our iPSC-derived astrocytes and neurons, with Krabbe astrocytes having ~2 fold higher C:16 galactosylceramide but lower C:18 galactosylceramide compared to controls. C:16, C24:1, and C:24 non-hydroxylated isoforms of glucosylceramide were all ~2–4 fold higher in Krabbe astrocytes compared to controls, and Krabbe cells also appeared to have a skewed ratio of C:16 to C:18, with higher C:16 and lower C:18 glucosylceramide compared to controls, which appeared to have slightly higher C:18 relative to C:16. The C:16 isoforms of hydroxylated galactosylceramide and glucosylceramide were the predominantly expressed isoforms in cultures from control and Krabbe donors, and were ~2–3 fold higher in Krabbe patent-derived astrocytes. It is worth noting that isoform length and hydroxylation state can affect the glycosphingolipid’s hydrophobic properties that result in differences in mobility and membrane localization [10], and that chain length-specific functions [80] and susceptibility to stimuli-induced apoptosis [81] have been observed. It is currently unclear if the shift in chain length to the shorter C:16 isoforms that we observed in the study are important to disease state, and additional work will be needed to understand whether glycosphingolipid chain length and hydroxylation differ in Krabbe patients and if these differences are significant in the context of disease progression.

We next explored the impact of Krabbe astrocytes on survival of neurons and microglia. Co-culturing neurons derived from two control donors onto established monolayers of astrocytes revealed a robust cytotoxic effect of the Krabbe astrocytes, with both Krabbe donor astrocyte lines negatively impacting the survival of neurons. Our finding is concordant with the results of Mangiameli et al. (2021) that showed a time-dependent reduction in Tuj1+ neurons in mixed neural cultures differentiated from a Krabbe iPSC line [48], although they do not address whether this observed phenotype is related to the astrocytes generated within the cultures through co-culture experiments as explored in our work. Dysfunction of astrocytes has been implicated in the neurodegenerative process for a number of lysosomal storage disorders [82]. For example, specific deletion of *Sumf1*, the gene associated with multiple sulfatase deficiency, in astrocytes induced degeneration of cortical neurons *in vivo* and *in vitro* [83]. Specific deletion of GalC in astrocytes has not been previously done, and given the data presented here such a study would be of interest. We also addressed whether direct cell-to-cell contact was required to observe the neurotoxicity of Krabbe astrocytes by applying astrocyte conditioned media to established neuron mono-cultures. Interestingly, Krabbe astrocyte conditioned media also appeared toxic to neurons, suggesting that, in part, a toxic intermediary is being secreted from Krabbe astrocytes. Notably, other groups have reported that exogenous psychosine exposure is toxic to human and murine neurons [22, 25]. Therefore, it is possible that in our system the psychosine accumulated in Krabbe astrocytes is in part secreted into the media and contributes to the observed neuronal toxicity. Alternatively, proteins secreted by Krabbe astrocytes, such as inflammatory cytokines may impact neuronal survival. Future proteomic or lipidomic studies examining the supernatant of Krabbe astrocytes may yield interesting information explaining the observed neurotoxicity and identify new targets for therapeutic intervention.

In stark contrast to the finding of cytotoxicity to neurons, we observed a greater number of control donor-derived microglia when co-cultured with Krabbe astrocytes compared to control astrocytes. The mechanisms as to how Krabbe astrocytes contributed to the increased number of microglia in our culture model was not the focus of the current studies, but the effect may be mediated by release of factors beneficial to microglial survival from astrocytes and/or influence via direct cell-to-cell interactions. As a proof-of-concept example, Takata et al., (2017) show that both co-culture with neurons or culture with neuron conditioned media is beneficial to iPSC-derived microglia survival, with direct co-culture being the most beneficial [84]. In this study we did not examine whether the beneficial effect of co-culture with Krabbe astrocytes on microglial survival required cell-cell contact or could be recapitulated with astrocyte-conditioned media alone. We also have not performed microglia-neuronal co-cultures, as this was beyond the scope of the current study, which focused on the role of Krabbe astrocytes. Microglia have been implicated in exacerbating the pathogenesis of Krabbe disease via mechanisms including promoting neuroinflammation and by the abnormal phagocytosis of myelin debris [85–87], and evidence suggests that microglia are the origin of the disease-namesake multinucleated globoid cells that appear around areas of demyelination [28, 29, 88]. To this end, psychosine-stimulated astrocytes release matrix metalloproteinase-3 (MMP-3) that has been shown to drive the formation of microglia into globoid cells [28], suggesting that cross talk between astrocytes and microglia are central to Krabbe disease.

It is important to address that an increased number of microglia might be beneficial in the setting of disease, and therefore the mechanisms leading to increased number of microglia in our culture system could provide insights into possible therapeutics. Alami et al. (2018) elegantly showed that astrocyte activation of NF-κb signaling promotes microglial proliferation in the SOD1 mutant mouse model of amyotrophic lateral sclerosis, which was beneficial in the early stages of disease by delaying onset of symptoms, but turned detrimental in the later stages of disease by accelerating disease progression [89]. The biphasic benefit of increased number of microglia was accompanied by a switch in microglia state, from an anti-inflammatory M2 state early in disease to a pro-inflammatory M1 state in the later stages [89]. In the context of Krabbe disease, Kondo et al. (2011) addressed the question as to whether the presence of microglia are beneficial by crossing the *twitcher* mouse with the macrophage deficient osteopetrotic mutant mouse, resulting in substantially fewer microglia and macrophages in the brain [90]. Results from their studies suggested that the lack of microglia accelerated disease progression, exacerbated disease phenotypes, and led to increased myelin debris in the white matter and impaired remyelination in the spinal cord [90]. Findings from these reports suggest that increased numbers of anti-inflammatory microglia may be beneficial in curbing disease progression. Therefore, it is possible that Krabbe astrocytes secrete factors that support microglia, thus reflecting their potential protective role. Future studies should aim to study the reactive state of microglia co-cultured with Krabbe astrocytes, as it may yield important insights into how astrocyte-microglia interactions are related to Krabbe disease pathogenesis.

In our final experiments, we addressed whether reduction of the elevated sphingolipids in Krabbe astrocytes had a beneficial effect on neuronal survival in co-culture. We show that small molecules can substantially reduce the accumulation of ceramide and glucosylceramide in Krabbe astrocytes, although we did not observe any benefit of these inhibitors on the number of neurons when co-cultured with Krabbe astrocytes. While we did not examine psychosine level in the GCS inhibitor experiment, as we would not expect a reduction with this compound, we did observe that psychosine levels were not reduced in Krabbe astrocytes following myriocin treatment. We would predict that inhibiting the ceramide biosynthesis pathway would reduce psychosine, or at least prevent further accumulation, by reducing the availability of its precursor galactosylceramide. Our result suggests that the psychosine present in the Krabbe cultures prior to myriocin treatment remains in the cells, as it is unable to be degraded due to deficiency in GALC, a protective function under normal conditions [7]. This is supported by the observation that the levels of psychosine don’t appear to further increase in the Day ~63 Krabbe astrocytes compared to their earlier Day 16 neuronal precursors (see S7 Fig). The changes we observed in gene expression of enzymes in the lipid biosynthesis pathway and the subsequent shunting away from psychosine towards glucosylceramide in Krabbe astrocytes, but not neurons, might be a compensatory mechanism that manifests over time in culture to limit the additional accumulation of psychosine. Together these data imply that substrate reduction approaches should target psychosine synthesis directly, and early in the disease state, rather than perturbations related to compensatory mechanisms. Indeed, recent evidence shows that inhibition of acid ceramidase, either genetically or with a small molecule, provides benefit on the survival of the Krabbe *twitcher* murine model [91]. Two reports have examined novel UGT8 inhibitors to block synthesize of psychosine precursor galactosylceramide, and showed reduction of brain psychosine, beneficial effects on neuroinflammation phenotypes, and extension of lifespans in Krabbe *twitcher* mice [92, 93]. Furthermore, studies using L-cycloserine, a serine palmitoyltransferase inhibitor, have shown benefit on survival and disease phenotypes in the *twitcher* model, but only when the compound is administered before the onset of symptoms [94, 95]. In the context of our findings, earlier treatment with myriocin might prove efficacious in our co-culture model if it were added prior to psychosine accumulation.

While our substrate reduction approach complements the strategies of the publications highlighted above, it is important to put our results in the context of reports suggesting that modulation of the sphingolipid pathway that is perturbed by GALC deficiency could be efficacious in the setting of Krabbe disease. Sphingosine-1-phosphate, a bioactive molecule within the sphingolipid metabolic pathway, signals through G-protein coupled receptors located on numerous cell types on the central and immune systems, and regulates diverse biological functions including apoptosis, cell growth, and inflammation, among others [32, 96, 97]. Agonism of the sphingosine-1-phosphate receptor (S1PR) with Fingolimod (FTY720) attenuated psychosine-induced cell death in cultured human astrocytes [32], psychosine-induced demyelination in mouse organotypic slice cultures [33] and importantly ameliorated demyelination, restored astrocyte and microglial reactivity, and extended lifespan in Krabbe *twitcher* mice [98]. The authors highlight that the beneficial effects of Fingolimod in Krabbe models could, in part, be mediated by its ability to modulate astrocyte reactivity and inflammatory cytokine release [32, 98]. With respect to our findings, we report that substrate reduction strategies targeting glucosylceramide accumulation did not produce robust changes in IL-6 levels in Krabbe astrocytes, which might suggest that we are not altering the inflammatory profile of these cells, and in the context of the findings discussed, could be related to our lack of treatment effect on the number of HuC/D+ neurons quantified in co-cultures. Further examining of the astrocyte inflammatory response by profiling additional cytokines would be helpful to answer the question of how broadly we are impacting their state. Furthermore, a future experiment could directly compare modulation of the S1PR to the substrate reduction approaches mentioned using our described phenotype in astrocyte-neuron co-cultures.

The results of our study need to be considered with respect to its limitations, in addition to those noted above. First, we only examined astrocytes generated from donors with infantile onset Krabbe disease, and both donors were homozygous for the large ~30 kb deletion in *GALC* found in Caucasians that results in near complete loss of enzyme activity [49]. Therefore, it is unclear as to whether astrocytes generated from donors harboring other disease-causing mutations, including those resulting in less severe later-onset forms of the disease, would display the same phenotypes identified in our study. Accordingly, it is challenging to interpret instances of between-line variability that we observed in some phenotypes, including IL-6 expression or psychosine levels following compound treatment, and it remains unclear whether these differences represent heterogeneity in patient phenotypes or *in vitro* assay artifacts. Examination of additional Krabbe lines will help elucidate the most relevant biological phenotypes in these cells. Indeed, the recent report examining differentiation of Krabbe donor iPSCs presented differences in lipidomic profiles and differentiation potential in patient lines with different mutations [48], highlighting the importance of profiling additional donor backgrounds. Second, we did not examine whether correcting expression of GALC in Krabbe astrocytes, perhaps by lentiviral-mediated overexpression of the gene in stem cells [99, 100] and subsequent differentiation into astrocyte lineage, would reverse the psychosine, ceramide, and glucosylceramide accumulation, compensatory expression changes in lipid synthesizing genes, and cytotoxicity to co-cultured neurons observed in our study. Without the *GALC* corrected cell lines it is difficult to interpret whether our findings are caused directly by deficient activity of GALC or compensatory changes in other enzymes. Third, while we assessed lipid accumulation in Krabbe neuronal cultures and noted psychosine accumulation, we did not further probe whether disease-related phenotypes could be observed in Krabbe neurons alone, as this was not the aim of the current study. However, others have noted neuron-specific phenotypes in neurons derived from human Krabbe donors [25] and the *twitcher* mouse [24, 27]. It would be of interest to explore how co-culture of Krabbe neurons with control astrocytes would behave, and if any defects observed in Krabbe neurons (for example, psychosine accumulation) could be corrected by co-culture with control astrocytes. Finally, we only explored perturbations in the lipid biosynthesis pathway directly surrounding psychosine. A multi-omics approach examining transcriptomic, lipidomic, and proteomic changes in Krabbe astrocytes would yield valuable insight into broad changes that might occur in disease-relevant cell types and offer novel targets for therapeutic interventions.

In conclusion, we show that astrocytes derived from Krabbe donors recapitulated many reported findings from human and rodent studies. We were able to uncover phenotypes relevant to the disease, including perturbed lipid biosynthesis and differential effects on neuron and microglia survival. Our results complement previous publications and further suggest that the contribution of astrocytes to the pathogenesis of Krabbe disease warrants further investigation.

## Supporting information

S1 Fig Validation of the large deletion within *GALC* in Krabbe fibroblasts. Prior to reprogramming, the ~30 kB deletion within *GALC* was confirmed in fibroblasts via qPCR by applying TaqMan selected probe and primer sets spanning specific exons. As expected, mRNA was detected using a probe spanning exons 3–4 in control and Krabbe fibroblasts. No detectable message was observed using probes spanning exons 13–14, 14–15, or 15–16, consistent with the deletion between exons 11–17 reported by the Coriell biobank.
(DOCX)

S2 Fig Qualitative assessment of Krabbe iPSCs ability to differentiate into neural cultures. Immunocytochemistry was used to assess the neural differentiation potential of Krabbe iPSCs prior to expansion and differentiation to astrocytes. *Top row*: No robust difference was observed in Krabbe iPSCs to generate cultures containing HuC/D+ (green) neurons and SOX2+ (orange) neural progenitors. *Middle row*: Control and Krabbe neural cultures contained Tuj1+ (green) neurites. A minor portion of the neurons in each of the cultures expressed dopaminergic-marker tyrosine hydroxylase (TH, orange). *Bottom row*: MAP2+ (orange) neurites were observed in control and Krabbe cultures. In all images, nuclei are counterstained with Hoechst and visualized in blue.
(DOCX)

S3 Fig Upregulation of *RIPK3* and *MT1E* in Krabbe astrocytes recapitulates findings in humans and in animal models of the disease. Basal expression of *RIKP3* and *MT1E* mRNA was upregulated in Krabbe astrocytes. These results are in line with published findings in human and rodent data sets showing elevated transcript expression in Krabbe disease. Data for individual donor subjects is shown as a box and whisker plot with median represented and errors bars indicating min/max values. Statistical analysis was conducted using t-tests on pooled data comparing control vs. Krabbe cells. **** p < 0.0001.
(DOCX)

S4 Fig mRNA expression of *IL-6*, *IL-8*, *IL-1β*, and *MCP-1 (CCL2)* following TNFα stimulation. Astrocytes from 2 healthy control donors and 2 Krabbe donors stimulated with 50 ng/mL TNFα for 24 hours. Significant effects with TNFα treatment, donor genotype, and interactions between the two were observed. TNFα treatment induced upregulation of mRNA levels of the four gene examined. Post-hoc analysis comparing expression following TNFα treatment revealed higher expression of *IL-6*, *IL-8*, *and IL-*1β, and lower expression of *MCP-1 (CCL2)* in Krabbe donors. Data for individual donor subjects is shown as a box and whisker plot with median represented and errors bars indicating min/max values, and is visualized relative to the mock condition of Control cells. Statistics were performed using two-way ANOVA with post-hoc test comparing expression values following TNF treatment. *** p < 0.001, **** p < 0.0001.
(DOCX)

S5 Fig Non-hydroxylated galactosylceramide and glucosylceramide species in iPSC-derived astrocytes. (A) The profile of non-hydroxylated galactosylceramide species in control and Krabbe astrocytes suggests that shorter carbon chain length species are the predominant isoforms. While no difference in total non-hydroxylated galactosylceramide was found (see Fig 2C), examination of the specific isoforms shows that the C16 species was elevated in Krabbe astrocytes, but the C18 isoform was more specific to control astrocytes. (B) The profile of non-hydroxylated glucosylceramide in control and Krabbe astrocytes suggests that the elevated levels of total glucosylceramide observed in Krabbe astrocytes is being driven by the short C16 and long C24:1 and C24 isoforms. Data showing isoform expression is depicted as mean ±SD.
(DOCX)

S6 Fig Hydroxylated (OH) galactosylceramide and glucosylceramide species in iPSC-derived astrocytes. (A) Hydroxylated galactosylceramide was higher in Krabbe astrocytes compared to controls. Examination of the species of OH-galactosylceramide expressed in our cell cultured revealed this increase was driven by C16 OH-galactosylceramide. (B) Hydroxylated glucosylceramide was significantly higher in Krabbe astrocytes compared to controls. This increase was also driven by C16 OH-glucosylceramide, which was the predominant isoform expressed in iPSC-derived astrocytes. Data for individual donor subjects is shown as a box and whisker plot with median represented and errors bars indicating min/max values. Data showing isoform expression is depicted as mean ±SD. Statistical analysis was conducted using t-tests on pooled data comparing control vs. Krabbe cells. ** p < 0.01.
(DOCX)

S7 Fig Comparison of total psychosine, galactosylceramide, and glucosylceramide lipid levels in iPSC-derived neuron and astrocyte mono-cultures from control and Krabbe donors. Cryopreserved neurons from 2 control and 2 Krabbe donors were seeded and cultured for 7 days prior to lipid analysis. Data and statistical significance from astrocytes is shown for comparison, and is identical to the data depicted in Fig 3. Astrocytes were generated from extended culture (~63–65 days) of the same neuronal cells. (A) Significantly elevated psychosine was detected in Krabbe neurons compared to Control neurons, where is was below the level of detection (t = 3.5, p = 0.007). The level of psychosine appeared comparable in Krabbe neurons and astrocytes. (B) A slight, but significant, elevation in non-hydroxylated galactosylceramide was observed in Krabbe neurons (t = 2.4, p = 0.04). (C-E) No significant differences were observed between control and Krabbe neurons in OH-galactosylceramide, glucosylceramide, or OH-glucosylceramide. Interestingly, both non-hydroxylated and hydroxylated glucosylceramide levels were substantially higher in Krabbe astrocytes compared to Krabbe neurons. Data for individual donor subjects is shown as a box and whisker plot with median represented and errors bars indicating min/max values. Statistical analysis was conducted using t-tests on pooled data comparing control vs. Krabbe cells. NS = not significant, * p < 0.05, ** p < 0.01, *** p < 0.001.
(DOCX)

S8 Fig Conditioned media from Krabbe astrocytes impedes neuronal survival. Neuronal media was conditioned on astrocytes from control and Krabbe donors for 7 days prior to being transferred onto already established neuron cultures from a healthy control donor for 96 hours. The live and dead cell fluorescent probes Calcein Green AM and NucRed Dead were used to quantify viability relative to the total number of nuclei identified via Hoechst staining (Note: Hoechst is not shown in the images). Quantification of the percent of live cells revealed a significant reduction when neurons were cultured in Krabbe astrocyte conditioned medium (p < 0.0001). Data was obtained from neurons grown in a 384 well plate, with 64 wells used for each conditioned media group. Data for individual donor subjects is shown as a box and whisker plot with median represented and errors bars indicating min/max values. Statistical analysis was conducted using t-tests on pooled data comparing control vs. Krabbe cells. **** p < 0.0001.
(DOCX)

S9 Fig Glucosylceramide synthase inhibition fails to rescue neuronal survival when co-cultured with Krabbe astrocytes. Astrocytes were thawed into 96 well assay plates. The following day media was replaced with fresh astrocyte media containing 18 nM (IC99) of an in-house glucosylceramide synthase inhibitor. Fresh media and compound were replaced every 3 days. After 9 days of treatment, cells were either harvested for lipid analysis via LC-MS-MS and IL-6 protein expression analysis via alphaLISA or used for a co-culture assay by seeding control neurons in neuronal media supplemented with GCS inhibitor for an additional 96 hours. (A-B) A simplified schematic of the ceramide biosynthetic pathway indicates the rationale for the experiment. Target engagement was validated following 9-day Inhibition of GCS by the reduced the levels of glucosylceramide and OH-glucosylceramide in the astrocyte cultures, including a substantial reduction in the Krabbe cells to near untreated healthy control levels. (C) IL-6 protein expression was not reduced in the astrocytes following GCS inhibitor treatment. (D-E) Co-culture of healthy control neurons with Krabbe astrocytes revealed no beneficial effect of GCS inhibition on neuronal survival. Data for individual donor subjects is shown as a box and whisker plot with median represented and errors bars indicating min/max values. Statistical analysis was determined as adjusted p < 0.05 and conducted using Two-way ANOVA with Bonferroni post hoc test comparing DMSO vs compound treated samples. ** p < 0.01, *** p < 0.001.
(DOCX)

S10 Fig Effects of SPTLC1 inhibition effects on OH-GlcCer and OH-GalCer in astrocytes. 6-day treatment with Myriocin reduced levels of hydroxylated (A) glucosylceramide and (B) galactosylceramide at all concentrations examined in iPSC-derived astrocytes from control and Krabbe donors. Data is shown as a box and whisker plot with median represented and errors bars indicating min/max values.
(DOCX)

S11 Fig SPTLC1 inhibition does not reduce Psychosine or IL-6 expression in Krabbe astrocytes. (A) 6-day treatment with Myriocin did not reduce Psychosine in Krabbe astrocytes. No change was observed in Krabbe 2 astrocytes. A significant upregulation was observed at the two lowest concentrations in Krabbe 1 astrocytes. (B) No significant change in IL-6 protein was detected in the supernatant of Krabbe 1 astrocytes treated with Myriocin, while slight, but significant reduction was seen at the 1 μM and 10 μM concentrations in Krabbe 2 astrocytes. Data is shown as a box and whisker plot with median represented and errors bars indicating min/max values. * p < 0.05, ** p < 0.01, **** p < 0.0001.
(DOCX)
