# Supplementary figures and images for: Human iPSC-derived astrocytes generated from donors with globoid cell leukodystrophy display phenotypes associated with disease
Source: PLoS One. 2022 Aug 3;17(8):e0271360. doi: 10.1371/journal.pone.0271360 (PMC9348679; doi:10.1371/journal.pone.0271360)

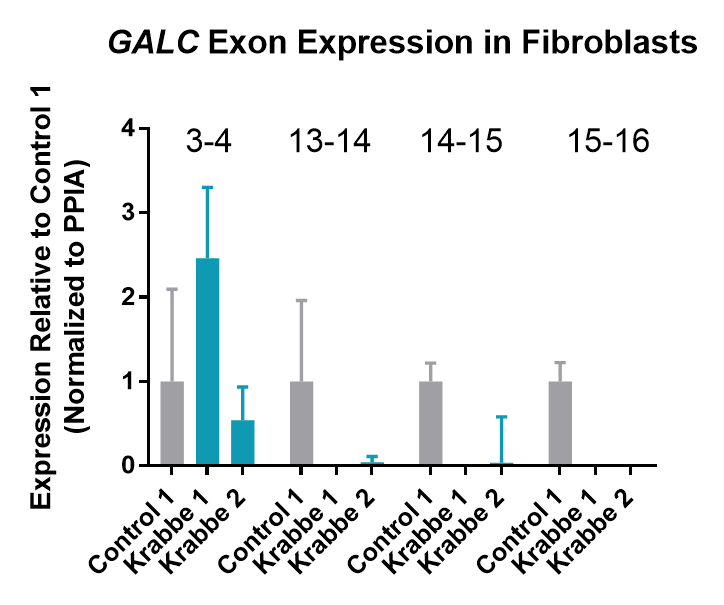

Supplement: S1 Fig — Prior to reprogramming, the ~30 kB deletion within GALC was confirmed in fibroblasts via qPCR by applying TaqMan selected probe and primer sets spanning specific exons. As expected, mRNA was detected using a probe spanning exons 3–4 in control and Krabbe fibroblasts. No detectable message was observed using probes spanning exons 13–14, 14–15, or 15–16, consistent with the deletion between exons 11–17 reported by the Coriell biobank. (DOCX) [file pone.0271360.s001.docx]

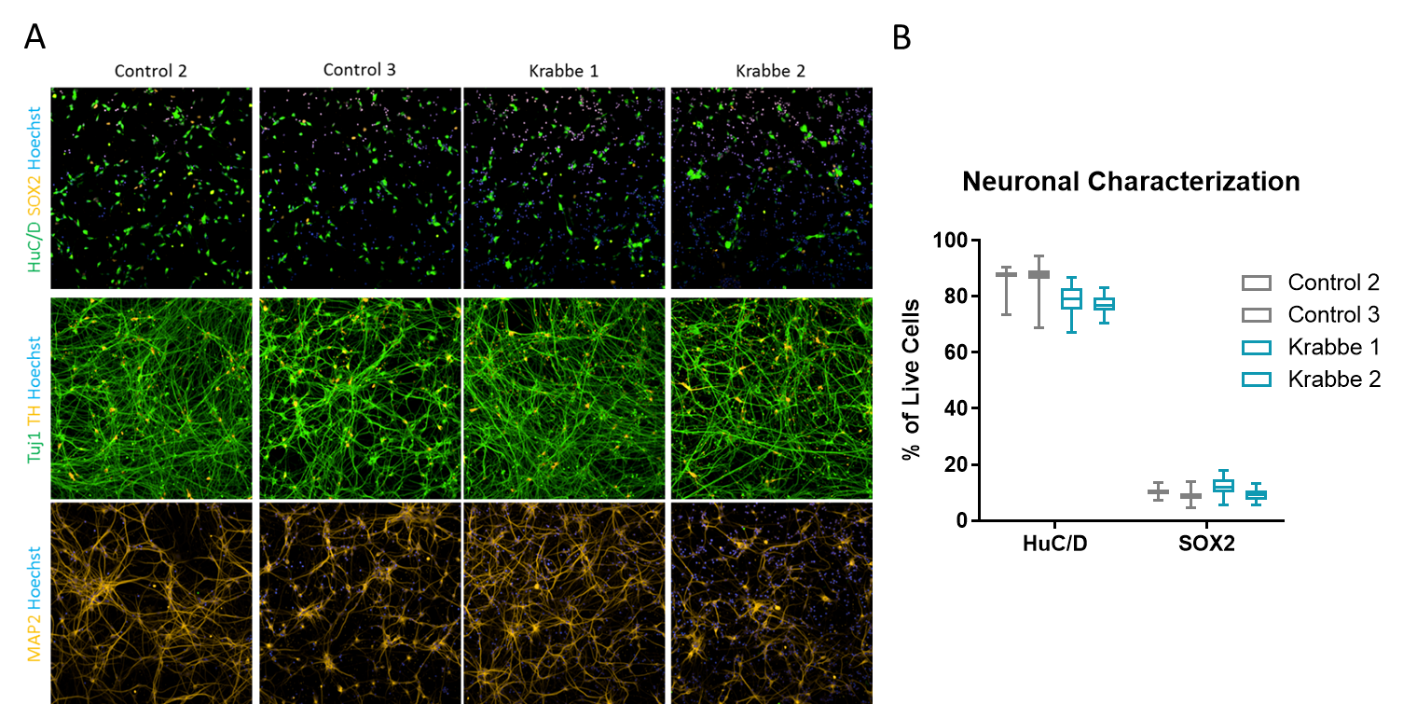

Supplement: S2 Fig — Immunocytochemistry was used to assess the neural differentiation potential of Krabbe iPSCs prior to expansion and differentiation to astrocytes. Top row: No robust difference was observed in Krabbe iPSCs to generate cultures containing HuC/D+ (green) neurons and SOX2+ (orange) neural progenitors. Middle row: Control and Krabbe neural cultures contained Tuj1+ (green) neurites. A minor portion of the neurons in each of the cultures expressed dopaminergic-marker tyrosine hydroxylase (TH, orange). Bottom row: MAP2+ (orange) neurites were observed in control and Krabbe cultures. In all images, nuclei are counterstained with Hoechst and visualized in blue. (DOCX) [file pone.0271360.s002.docx]

**
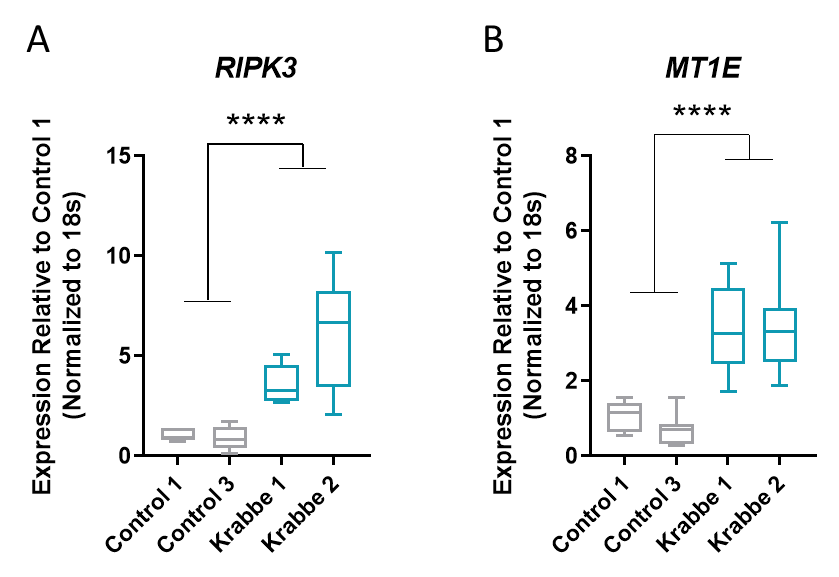
**

Supplement: S3 Fig — Basal expression of RIKP3 and MT1E mRNA was upregulated in Krabbe astrocytes. These results are in line with published findings in human and rodent data sets showing elevated transcript expression in Krabbe disease. Data for individual donor subjects is shown as a box and whisker plot with median represented and errors bars indicating min/max values. Statistical analysis was conducted using t-tests on pooled data comparing control vs. Krabbe cells. **** p < 0.0001. (DOCX) [file pone.0271360.s003.docx]

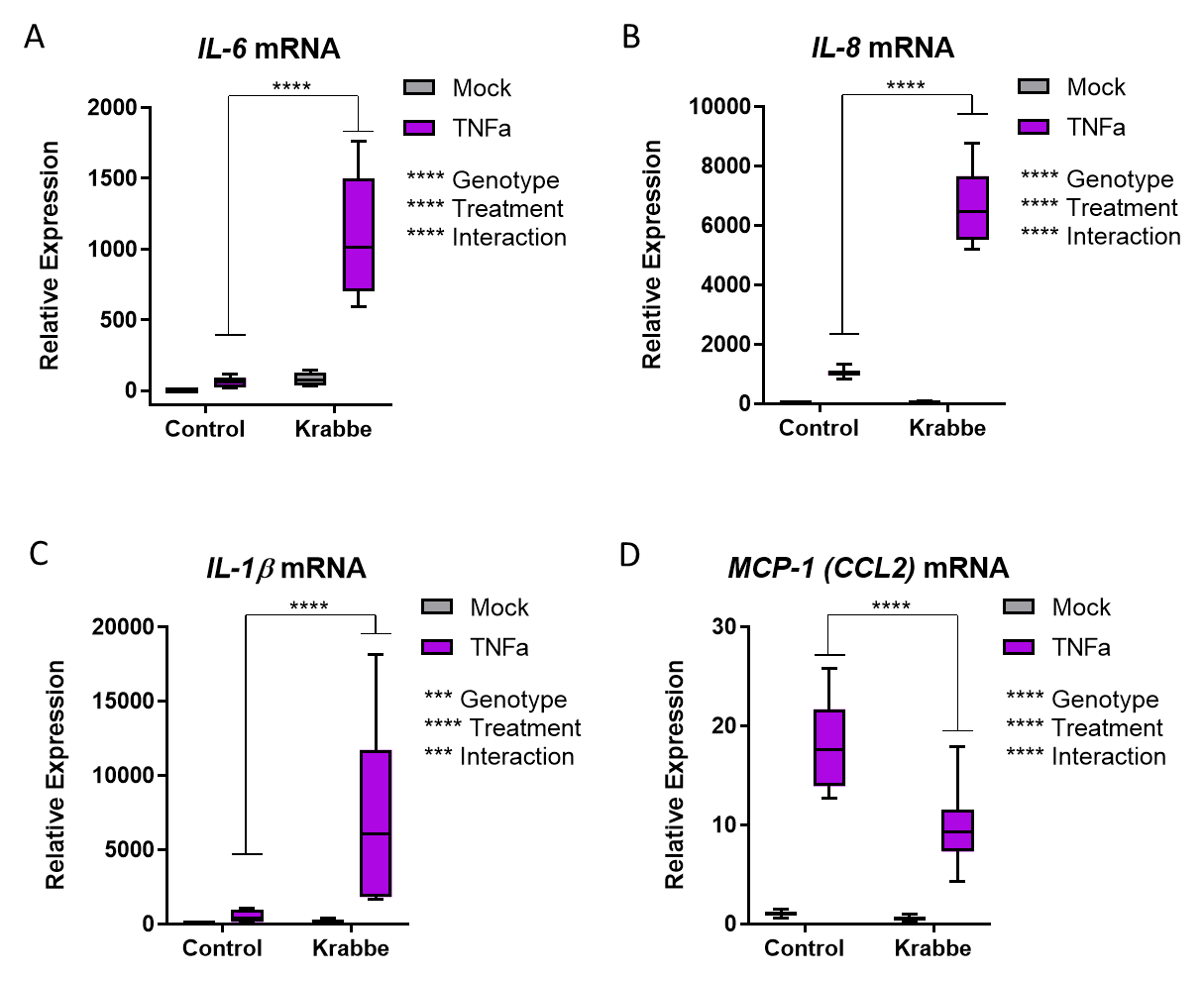

Supplement: S4 Fig — Astrocytes from 2 healthy control donors and 2 Krabbe donors stimulated with 50 ng/mL TNFα for 24 hours. Significant effects with TNFα treatment, donor genotype, and interactions between the two were observed. TNFα treatment induced upregulation of mRNA levels of the four gene examined. Post-hoc analysis comparing expression following TNFα treatment revealed higher expression of IL-6, IL-8, and IL-1β, and lower expression of MCP-1 (CCL2) in Krabbe donors. Data for individual donor subjects is shown as a box and whisker plot with median represented and errors bars indicating min/max values, and is visualized relative to the mock condition of Control cells. Statistics were performed using two-way ANOVA with post-hoc test comparing expression values following TNF treatment. *** p < 0.001, **** p < 0.0001. (DOCX) [file pone.0271360.s004.docx]

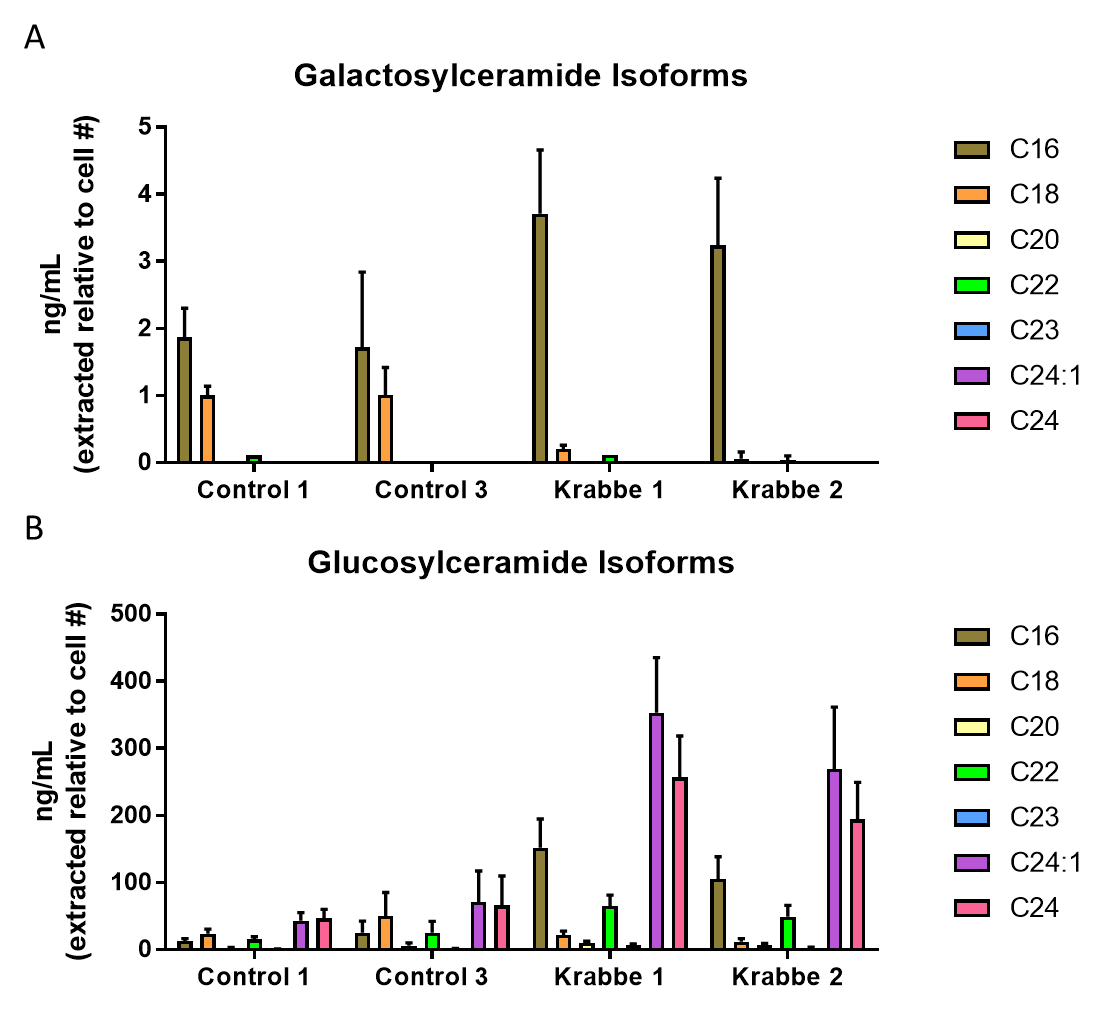

Supplement: S5 Fig — (A) The profile of non-hydroxylated galactosylceramide species in control and Krabbe astrocytes suggests that shorter carbon chain length species are the predominant isoforms. While no difference in total non-hydroxylated galactosylceramide was found (see Fig 2C), examination of the specific isoforms shows that the C16 species was elevated in Krabbe astrocytes, but the C18 isoform was more specific to control astrocytes. (B) The profile of non-hydroxylated glucosylceramide in control and Krabbe astrocytes suggests that the elevated levels of total glucosylceramide observed in Krabbe astrocytes is being driven by the short C16 and long C24:1 and C24 isoforms. Data showing isoform expression is depicted as mean ±SD. (DOCX) [file pone.0271360.s005.docx]

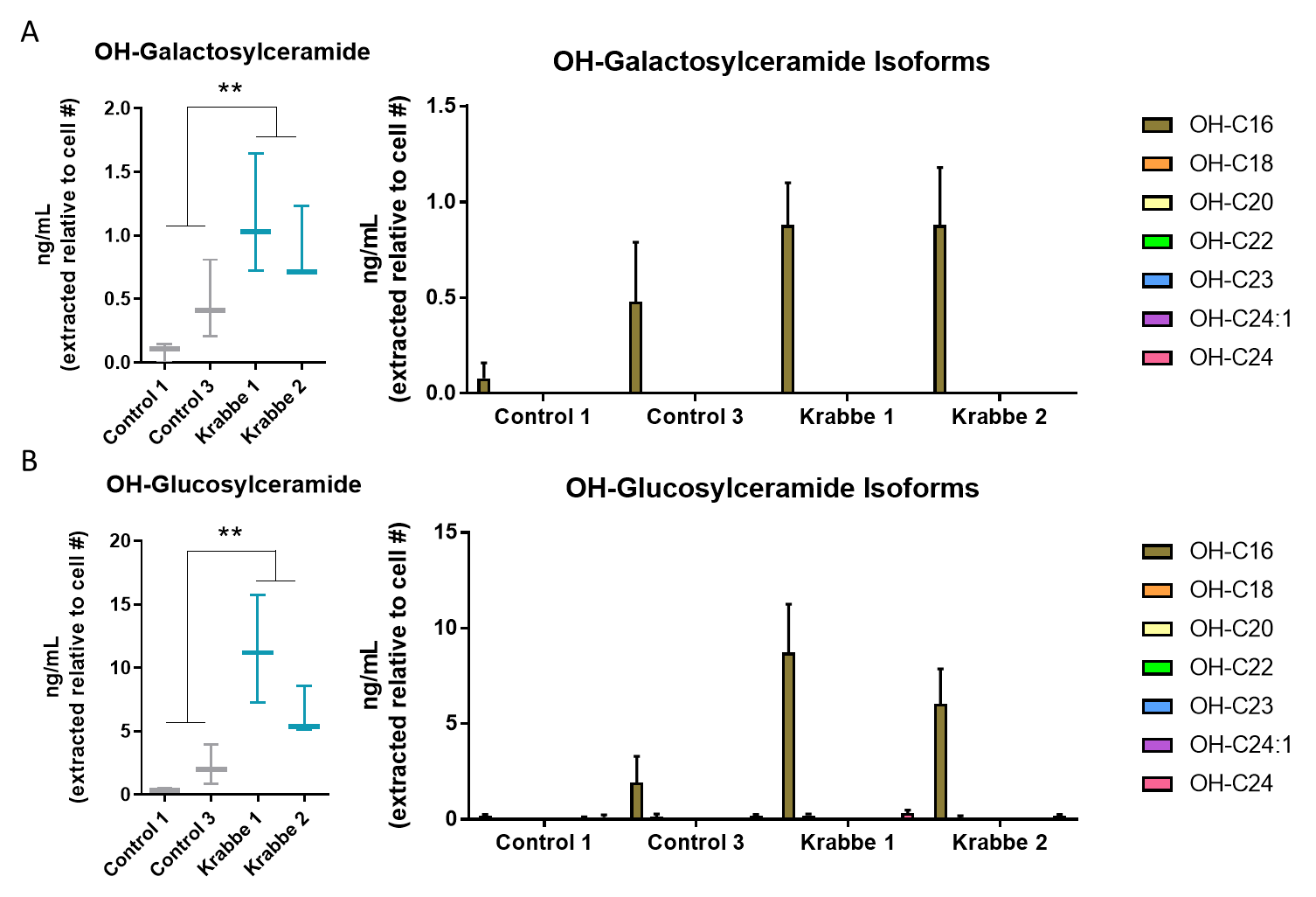

Supplement: S6 Fig — (A) Hydroxylated galactosylceramide was higher in Krabbe astrocytes compared to controls. Examination of the species of OH-galactosylceramide expressed in our cell cultured revealed this increase was driven by C16 OH-galactosylceramide. (B) Hydroxylated glucosylceramide was significantly higher in Krabbe astrocytes compared to controls. This increase was also driven by C16 OH-glucosylceramide, which was the predominant isoform expressed in iPSC-derived astrocytes. Data for individual donor subjects is shown as a box and whisker plot with median represented and errors bars indicating min/max values. Data showing isoform expression is depicted as mean ±SD. Statistical analysis was conducted using t-tests on pooled data comparing control vs. Krabbe cells. ** p < 0.01. (DOCX) [file pone.0271360.s006.docx]

**
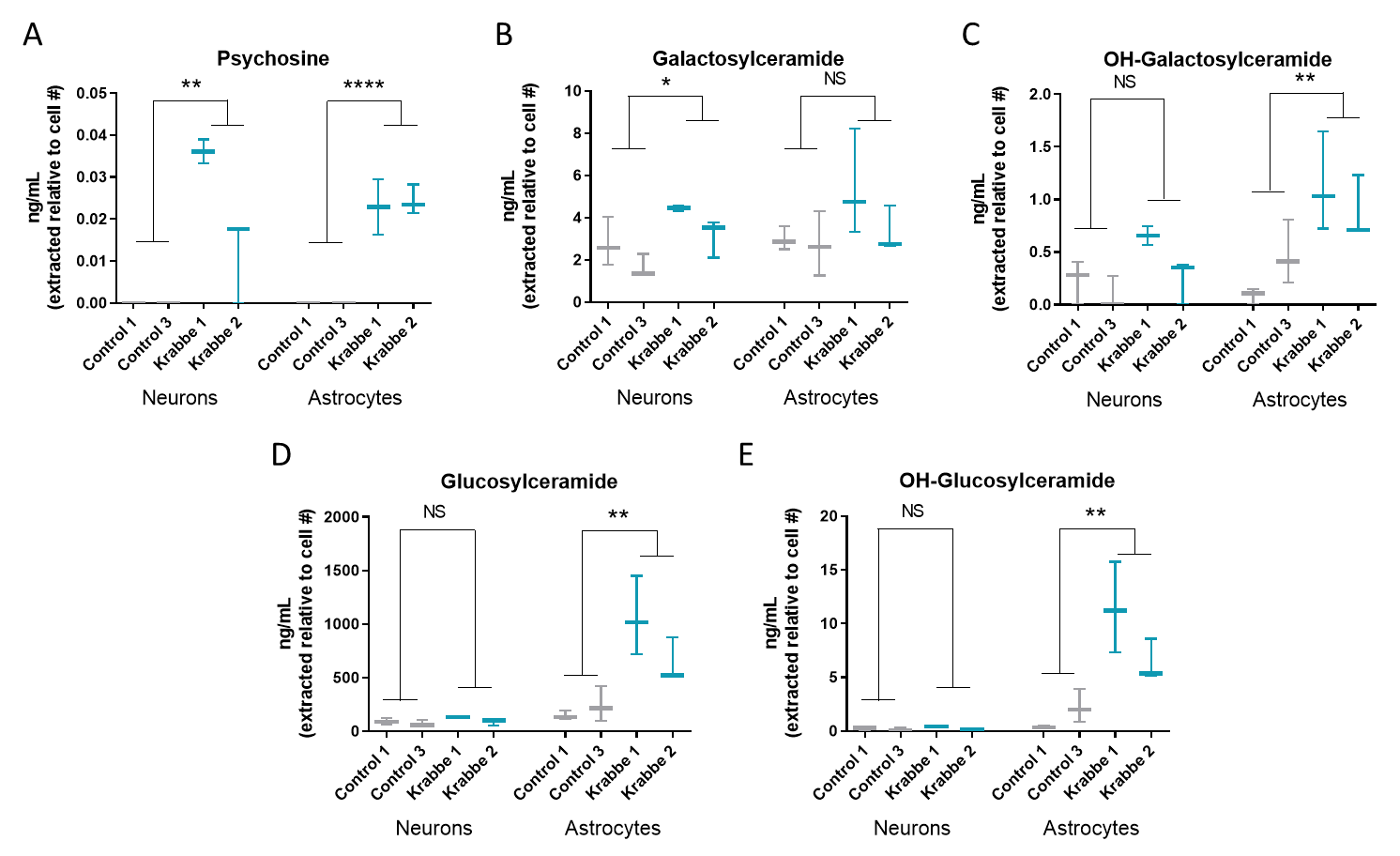
**

Supplement: S7 Fig — Cryopreserved neurons from 2 control and 2 Krabbe donors were seeded and cultured for 7 days prior to lipid analysis. Data and statistical significance from astrocytes is shown for comparison, and is identical to the data depicted in Fig 3. Astrocytes were generated from extended culture (~63–65 days) of the same neuronal cells. (A) Significantly elevated psychosine was detected in Krabbe neurons compared to Control neurons, where is was below the level of detection (t = 3.5, p = 0.007). The level of psychosine appeared comparable in Krabbe neurons and astrocytes. (B) A slight, but significant, elevation in non-hydroxylated galactosylceramide was observed in Krabbe neurons (t = 2.4, p = 0.04). (C-E) No significant differences were observed between control and Krabbe neurons in OH-galactosylceramide, glucosylceramide, or OH-glucosylceramide. Interestingly, both non-hydroxylated and hydroxylated glucosylceramide levels were substantially higher in Krabbe astrocytes compared to Krabbe neurons. Data for individual donor subjects is shown as a box and whisker plot with median represented and errors bars indicating min/max values. Statistical analysis was conducted using t-tests on pooled data comparing control vs. Krabbe cells. NS = not significant, * p < 0.05, ** p < 0.01, *** p < 0.001. (DOCX) [file pone.0271360.s007.docx]

**
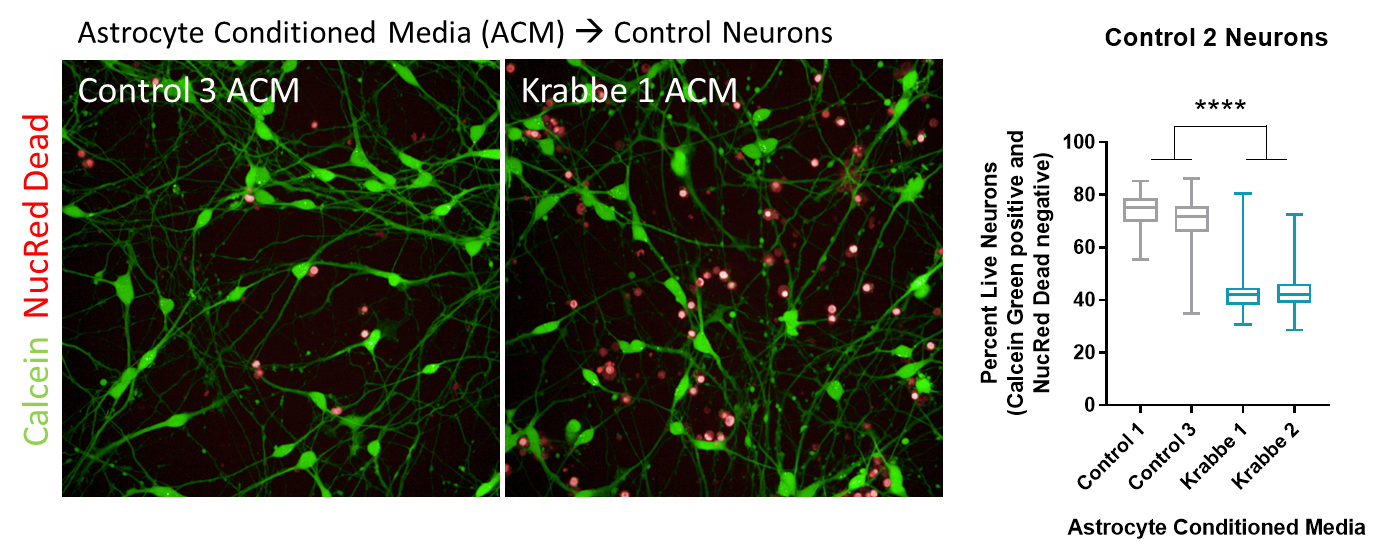
**

Supplement: S8 Fig — Neuronal media was conditioned on astrocytes from control and Krabbe donors for 7 days prior to being transferred onto already established neuron cultures from a healthy control donor for 96 hours. The live and dead cell fluorescent probes Calcein Green AM and NucRed Dead were used to quantify viability relative to the total number of nuclei identified via Hoechst staining (Note: Hoechst is not shown in the images). Quantification of the percent of live cells revealed a significant reduction when neurons were cultured in Krabbe astrocyte conditioned medium (p < 0.0001). Data was obtained from neurons grown in a 384 well plate, with 64 wells used for each conditioned media group. Data for individual donor subjects is shown as a box and whisker plot with median represented and errors bars indicating min/max values. Statistical analysis was conducted using t-tests on pooled data comparing control vs. Krabbe cells. **** p < 0.0001. (DOCX) [file pone.0271360.s008.docx]

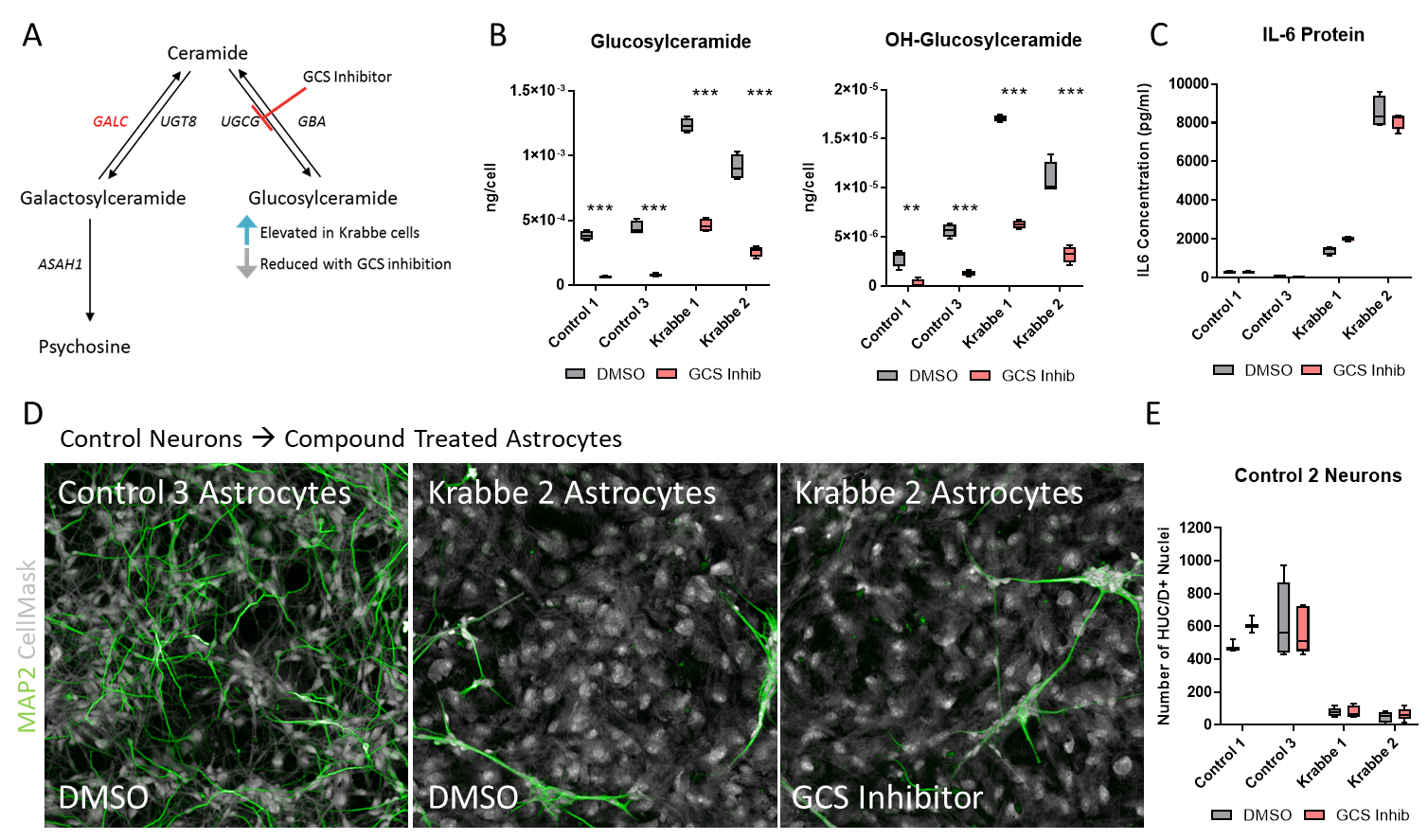

Supplement: S9 Fig — Astrocytes were thawed into 96 well assay plates. The following day media was replaced with fresh astrocyte media containing 18 nM (IC99) of an in-house glucosylceramide synthase inhibitor. Fresh media and compound were replaced every 3 days. After 9 days of treatment, cells were either harvested for lipid analysis via LC-MS-MS and IL-6 protein expression analysis via alphaLISA or used for a co-culture assay by seeding control neurons in neuronal media supplemented with GCS inhibitor for an additional 96 hours. (A-B) A simplified schematic of the ceramide biosynthetic pathway indicates the rationale for the experiment. Target engagement was validated following 9-day Inhibition of GCS by the reduced the levels of glucosylceramide and OH-glucosylceramide in the astrocyte cultures, including a substantial reduction in the Krabbe cells to near untreated healthy control levels. (C) IL-6 protein expression was not reduced in the astrocytes following GCS inhibitor treatment. (D-E) Co-culture of healthy control neurons with Krabbe astrocytes revealed no beneficial effect of GCS inhibition on neuronal survival. Data for individual donor subjects is shown as a box and whisker plot with median represented and errors bars indicating min/max values. Statistical analysis was determined as adjusted p < 0.05 and conducted using Two-way ANOVA with Bonferroni post hoc test comparing DMSO vs compound treated samples. ** p < 0.01, *** p < 0.001. (DOCX) [file pone.0271360.s009.docx]

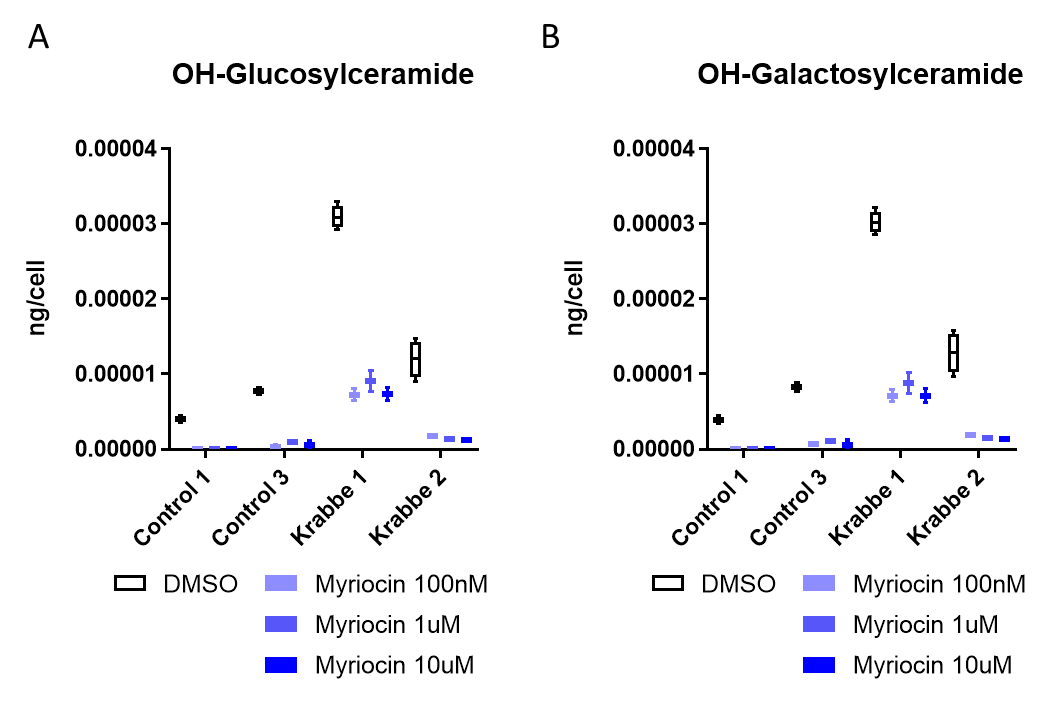

Supplement: S10 Fig — 6-day treatment with Myriocin reduced levels of hydroxylated (A) glucosylceramide and (B) galactosylceramide at all concentrations examined in iPSC-derived astrocytes from control and Krabbe donors. Data is shown as a box and whisker plot with median represented and errors bars indicating min/max values. (DOCX) [file pone.0271360.s010.docx]

**
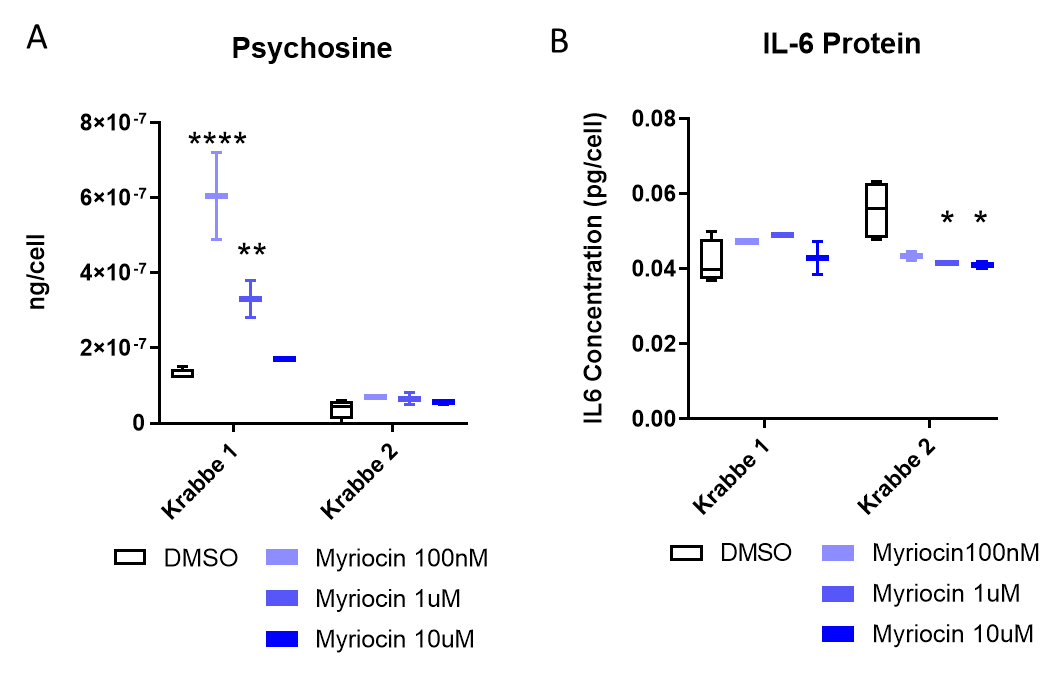
**

Supplement: S11 Fig — (A) 6-day treatment with Myriocin did not reduce Psychosine in Krabbe astrocytes. No change was observed in Krabbe 2 astrocytes. A significant upregulation was observed at the two lowest concentrations in Krabbe 1 astrocytes. (B) No significant change in IL-6 protein was detected in the supernatant of Krabbe 1 astrocytes treated with Myriocin, while slight, but significant reduction was seen at the 1 μM and 10 μM concentrations in Krabbe 2 astrocytes. Data is shown as a box and whisker plot with median represented and errors bars indicating min/max values. * p < 0.05, ** p < 0.01, **** p < 0.0001. (DOCX) [file pone.0271360.s011.docx]
